# Heterogeneity of Human Neutrophil CD177 Expression Results from *CD177P1* Pseudogene Conversion

**DOI:** 10.1371/journal.pgen.1006067

**Published:** 2016-05-26

**Authors:** Zuopeng Wu, Rong Liang, Thomas Ohnesorg, Vicky Cho, Wesley Lam, Walter P. Abhayaratna, Paul A. Gatenby, Chandima Perera, Yafei Zhang, Belinda Whittle, Andrew Sinclair, Christopher C. Goodnow, Matthew Field, T. Daniel Andrews, Matthew C. Cook

**Affiliations:** 1 Translational Research Unit, Canberra Hospital, Woden, Australian Capital Territory, Australia; 2 Clinical Trials Unit, Canberra Hospital, Woden, Australian Capital Territory, Australia; 3 Australian Phenomics Facility, Australian National University, Australian Capital Territory, Australia; 4 Murdoch Children’s Research Institute, Department of Paediatrics, The University of Melbourne, The Royal Children's Hospital, Melbourne, Victoria, Australia; 5 Department of Immunology, The John Curtin School of Medical Research, Australian National University, Acton, Australian Capital Territory, Australia; 6 Department of Immunology, Canberra Hospital, Woden, Australian Capital Territory, Australia; 7 Department of Rheumatology, Canberra Hospital, Woden, Australian Capital Territory, Australia; Ospedale San Pietro Fatebenefratelli, ITALY

## Abstract

Most humans harbor both CD177^neg^ and CD177^pos^ neutrophils but 1–10% of people are CD177^null^, placing them at risk for formation of anti-neutrophil antibodies that can cause transfusion-related acute lung injury and neonatal alloimmune neutropenia. By deep sequencing the *CD177* locus, we catalogued *CD177* single nucleotide variants and identified a novel stop codon in CD177^null^ individuals arising from a single base substitution in exon 7. This is not a mutation in *CD177* itself, rather the CD177^null^ phenotype arises when exon 7 of *CD177* is supplied entirely by the CD177 pseudogene (*CD177P1*), which appears to have resulted from allelic gene conversion. In CD177 expressing individuals the *CD177* locus contains both *CD177P1* and *CD177* sequences. The proportion of CD177^hi^ neutrophils in the blood is a heritable trait. Abundance of CD177^hi^ neutrophils correlates with homozygosity for *CD177* reference allele, while heterozygosity for ectopic *CD177P1* gene conversion correlates with increased CD177^neg^ neutrophils, in which both *CD177P1* partially incorporated allele and paired intact *CD177* allele are transcribed. Human neutrophil heterogeneity for CD177 expression arises by ectopic allelic conversion. Resolution of the genetic basis of CD177^null^ phenotype identifies a method for screening for individuals at risk of CD177 isoimmunisation.

## Author Summary

Expression of the neutrophil-specific antigen CD177 varies across the population. 1–10% of humans are CD177^null^. CD177^pos^ neonates born to CD177^null^ mothers are susceptible to alloimmune neutropenia. Interestingly, CD177^pos^ and CD177^neg^ populations of neutrophils often exist together within individuals. The reasons for heterogeneous CD177 expression are not well understood. We deep sequenced the *CD177* locus in individuals with different levels of CD177 expression, catalogued *CD177* single nucleotide variants, and identified a premature stop codon that causes lack of CD177 expression. Comparison of messenger RNA from neutrophils with genomic *CD177* DNA identified significant sequence similarity with *CD177P1* pseudogene, which probably explains existing misannotation in public databases, but also explains susceptibility to cross-over errors. Indeed, we report that the stop codon responsible for the CD177^null^ phenotype arises when exon 7 of *CD177* gene is supplied entirely by *CD177P1* by gene conversion. We also show that the proportion of CD177^hi^ neutrophil numbers within individuals is a heritable trait, determined by the proportion of intact *CD177* and converted *CD177* alleles. Furthermore, within individuals, *CD177* gene is differentially transcribed in CD177^neg^ and CD177^hi^ neutrophils. Our work resolves the genetic basis of CD177 phenotype and identifies a method for screening individuals at risk of CD177 isoimmunisation.

## Introduction

CD177 (also known as neutrophil specific antigen B1 [NB1], human neutrophil antigen 2a [HNA-2a], and polycythemia rubra vera 1 [PRV1]) is a 56–64 kDa protein belonging to the Ly-6 family [1, 2], and is expressed exclusively on neutrophils by glycosylphosphatidylinositol (GPI)-linkage [3, 4]. CD177 expression is heterogeneous within the human population. 1–10% of people are CD177^null^, while the remainder harbor neutrophils that are bi- or tri-modal for CD177 expression [4–6].

Heterogeneity for neutrophil surface antigen expression within the population results in susceptibility to alloantibody formation due to loss of acquired immunological tolerance. Transplacental passage or transfusion of neutrophil antibodies have been implicated in severe neonatal alloimmune neutropenia (NAN) [7] and transfusion related acute lung injury (TRALI) [8, 9]. Polymorphic human neutrophil antigens (HNA) include CD16 or FcγRIIIb (HNA-1a, -1b, -1c and -1d, encoded by *FCGR3B*) [10], CD177 (HNA-2a, encoded by *CD177*) [11, 12], choline transporter-like protein 2 (HNA-3a) [13–15], CD11b (HNA-4a) and CD11a (HNA-5a) [16–19]. In some cases, the genetic polymorphisms that account for alloantigenicity have been resolved. For example, three separate alleles of *FCGR3B* (designated HNA-1a, -1b, and -1c) appear to account for CD16 alloantigenicity, and the different epitopes of each defined isoform are specified by variations of five amino acid exchanges [10, 20–22]. Similarly, single amino acid substitutions account for HNA-3a antigenicity [13, 14, 23]. Absence of HNA-1a, -1b, -1c, -1d, HNA-3a, HNA-4a and HNA-5a has been associated with formation of maternal alloantibodies [24, 25]. CD177 deficiency has also been shown to result in development of maternal alloantibodies that cause neonatal alloimmune neutropenia [11]. In addition, CD177 is pertinent to systemic vasculitis, since one of the principal autoantigens, proteinase 3, is a constituent of primary granules, but is exposed on the neutrophil surface in association with CD177 [26, 27]. Complete elucidation of the genetic basis of neutrophil alloantigenic variation is an important goal, since testing for neutrophil antibodies is technically challenging and limited in current clinical practice [28]. By contrast, identification of the genetic basis of antigen expression, particularly for absence of antigen, permits screening individuals at risk of generating specific antibodies in these disease settings [29].

Variation in CD177 expression has been the subject of previous investigations (S1 Table). Analysis of mRNA amplified from neutrophils of two CD177^null^ donors showed two separate RNA insertions. In one case, there was an intronic fragment inserted into exon 6, and in the other an alternative 5’ end splicing donor of exon 4. Both were postulated to cause loss of CD177 expression by introducing an in-frame premature stop codon [30, 31] (S1 Fig). Further investigation revealed an association between certain *CD177* single nucleotide variations (SNVs) and different CD177 phenotypes, although the mechanism to account for these effects was not elucidated, and the association was insufficient to permit diagnostic testing [32, 33]. More recently, a SNV of cDNA829A>T mutation that introduces a stop codon was identified in *CD177* as a cause for loss of CD177 expression [34].

We set out to determine the genetic basis of inter- and intra-individual CD177 phenotypes, by deep sequencing neutrophil-derived genomic DNA across the *CD177* locus. Taking this approach, we confirmed the stop codon identified by Li et al in CD177^null^ individuals, but discovered that variation arises when exon 7 of *CD177* gene is supplied entirely by allelic conversion with the CD177 pseudogene (*CD177P1*), which comprises sequences homolog of *CD177* exons 4–9 on the minus strand. This variant is present within the germline rather than somatically acquired within neutrophils. Individuals who are homozygous for *CD177* have higher CD177 expression, whereas individuals with ectopic *CD177P1* exon 7 conversion have larger proportions of CD177^neg^ neutrophils in the blood. We demonstrated that CD177 is a heritable trait determined by the ratio of *CD177*/*CD177P1* alleles, and uncovered distinctive CD177 transcription in CD177^neg^ and CD177^hi^ neutrophils within the same individual. These findings resolve the basis of interindividual (CD177^null^ versus CD177 expressing) and intra-individual (CD177^neg^ versus CD177^hi^ subsets) CD177 expression, and identify a method for screening for individuals at risk of CD177 isoimmunisation.

## Results

### CD177 heterogeneity

**Fig 1:**
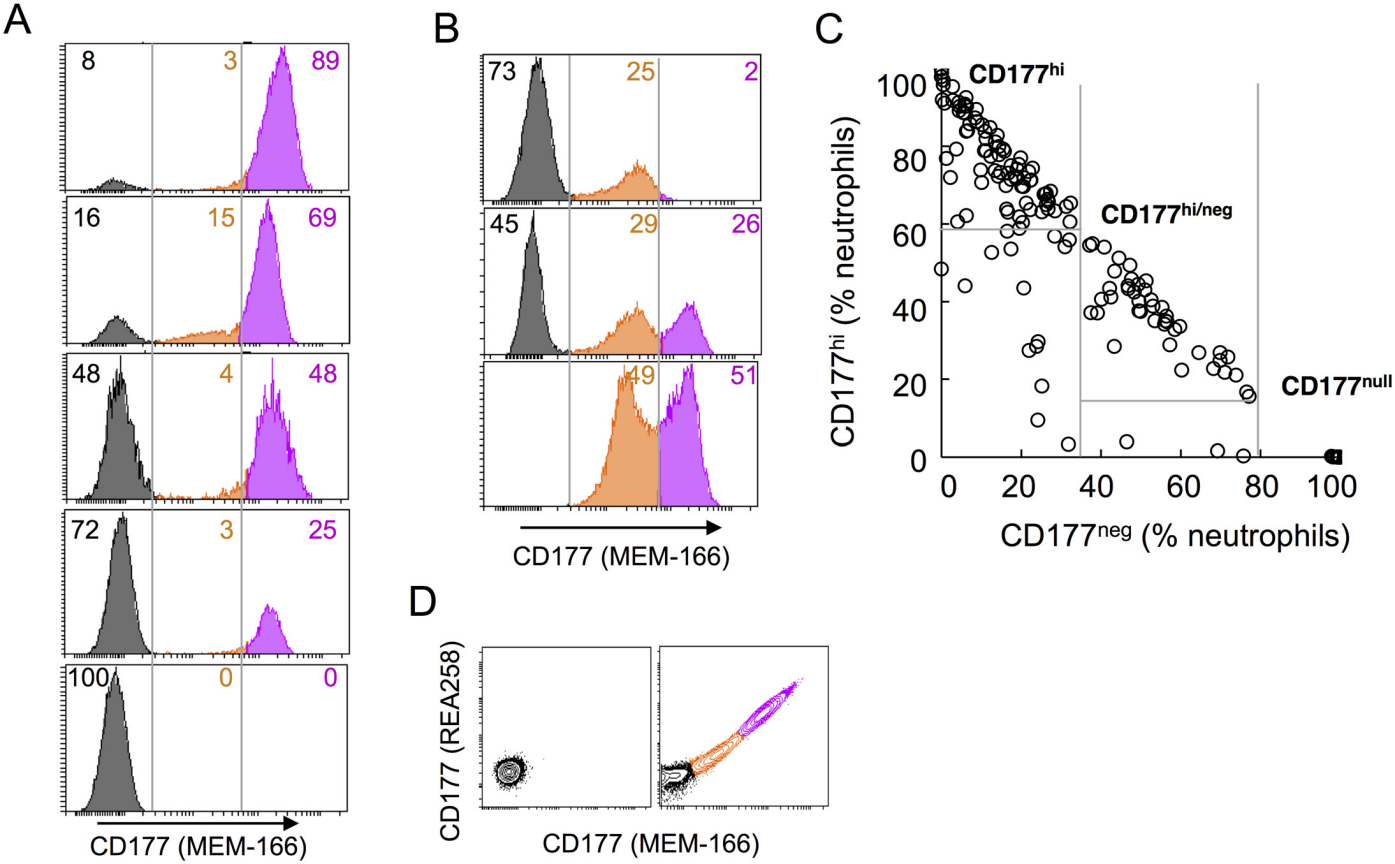
CD177 expression in neutrophils. **A.** Neutrophils of 5 subjects were gated on CD66b^+^ cells and analysed for surface expression of CD177. Three neutrophil subsets defined by CD177 expression were shown: negative (neg) in black, intermediate (int) in orange and high (hi) in pink, percentages of each subset were indicated as numbers in the same colour. **B**. Atypical CD177 expression with CD177^int^ subset >20% of neutrophils. **C.** A dot plot showing percentages of CD177^hi^ versus CD177^neg^ of total neutrophils in healthy subjects, each dot represents one subject. Three major phenotypic groups were marked as CD177^hi^, CD177^hi/neg^, and CD177^null^. **D.** Contour plots showing neutrophils from a CD177^null^ subject (left) and a tri-model CD177 expressing donor (right), co-stained with two CD177 specific monoclonal antibodies (MEM-166 and REA258).

Our discovery cohort comprised 40 patients with systemic vasculitis (cohort 1), and emerged as part of investigation of expression of vasculitis-associated autoantigens (proteinase 3 (PR3), myeloperoxidase, and the associated alloantigen CD177). Our study population was made up of individuals of European, Asian and Australian self-reported ethnicity (S2A Fig). Consistent with previous reports [33, 35], we identified three neutrophil populations according to CD177 expression: negative (neg), intermediate (int) and high (hi). The majority of individuals were bi-modal for CD177 (CD177^hi^ and CD177^neg^) (Fig 1A), while some individuals harbor a substantial proportion (>20%) of neutrophils expressing CD177 at intermediate levels (Fig 1B). In a larger cohort (n = 535) of healthy donors (cohort 2), 65.4% of the subjects’ neutrophils were predominantly CD177^hi^ (Fig 1C), while in 24.7% of the cohort, the distribution of CD177^hi^ and CD177^neg^ were similar (CD177^hi/neg^). 2.6% (n = 14) were found to be CD177^null^. A similar prevalence of CD177 phenotypes were observed in both cohorts (S2B Fig). We found that CD177 phenotypes are stable within individuals over six months (S2C, S2D and S2E Fig). Flow cytometric analysis using two different CD177 monoclonal antibodies (MEM-166 and REA258) yielded similar results, indicating absence of CD177 expression rather than modification of a CD177 epitope in CD177^null^ (Fig 1D).

### Two exons with enriched SNV density and a novel stop codon variation in *CD177* gene

**Fig 2:**
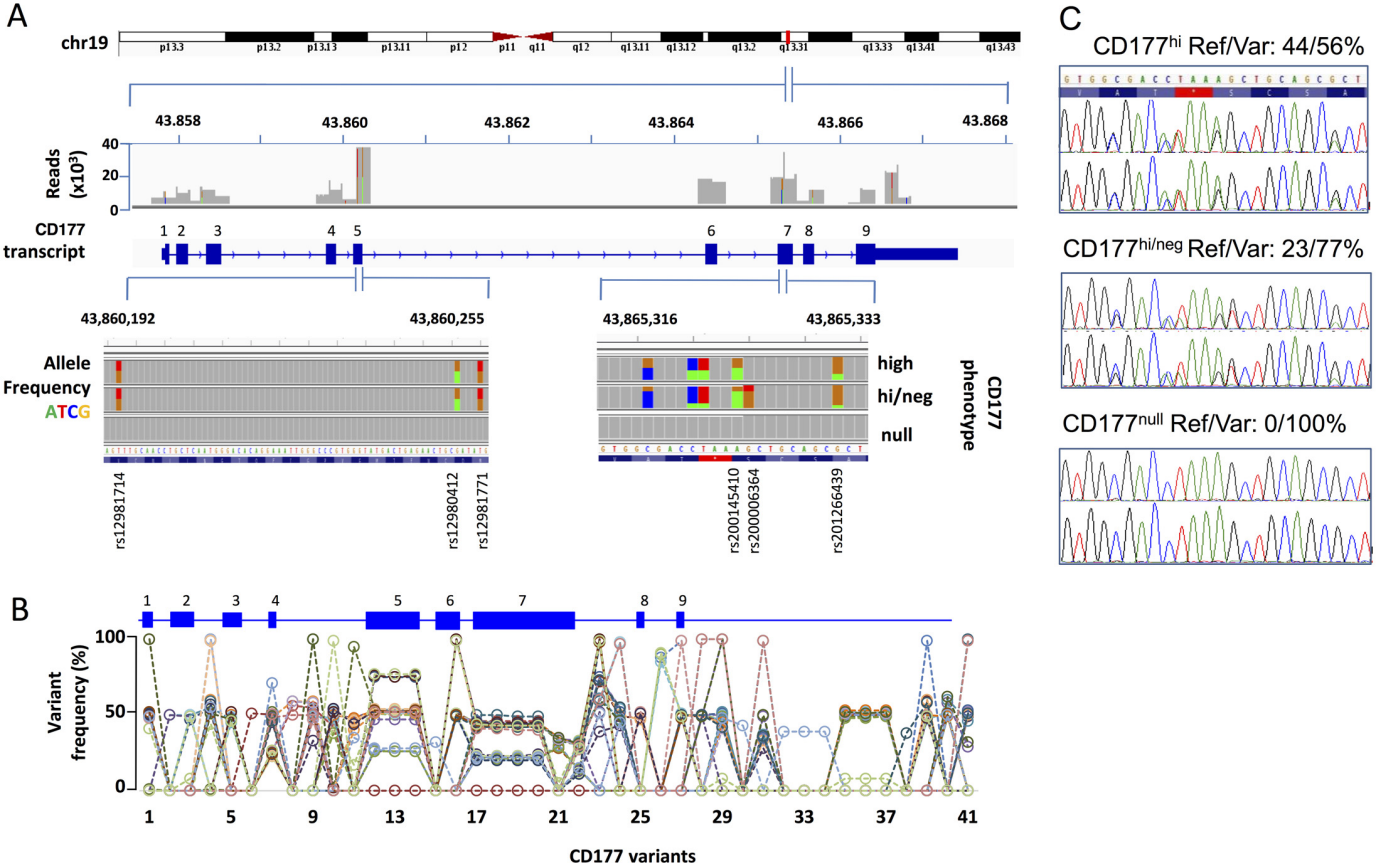
Two exons of enriched SNP density and a novel stop codon variation in *CD177* gene. **A**. Summary of deep sequencing of *CD177* gene (located at 43.8Mb, band q13.2, of the forward strand of chromosome 19 in human genome 19, 43.3 Mb and q13.31 for hg38). Amplicons covering the entire coding sequence of 9 exons were sequenced. 100% coverage was obtained at indicated read depths. 24 low frequency SNPs were found in exon adjacent non-coding regions and 17 polymorphisms were identified in *CD177* coding sequences. Representative allelic frequencies of nine SNPs in exon 5 and 7 are displayed according to CD177 phenotypes. Graphs were generated from Integrative Genomics Viewer from Broad Institute referenced on hg19. **B**. SNV frequency of 41 variants (called from hg19) in cohort 1. Each dashed line represents genotypes from a single individual. A schematic *CD177* gene structure is shown of variants within *CD177*. **C**. Sanger sequencing of *CD177* exon 7 in DNA isolated from neutrophils (upper panel) and saliva (lower panel) from three individuals same as shown in **A**. Reference (Ref) and variant (Var) read frequencies derived from deep sequencing and CD177 neutrophil phenotypes for each subject were indicated on the top of each panel.

We deep sequenced *CD177* in cohort 1. To ensure that we did not miss somatic mutations we isolated genomic DNA using a custom capture array specifically from neutrophils purified from each subject. Loci containing all nine *CD177* exons were isolated and deep sequenced (>9,000x) (Fig 2A). We identified 41 SNVs, including 17 in the coding regions (Fig 2A and 2B and Table 1). These included common non-synonymous SNVs in exon 5 (rs12981714, rs12980412 and rs12981771) in 39/40 subjects. We also identified three non-synonymous SNVs in exon 7 (rs200145410, rs200006364 and rs201266439) and three novel variants located within five nucleotides of each other in all 40 subjects. One of these novel variants (genomic location 19:43,361,169, c.787A>T, g.7497A>T in hg38) changes a lysine codon (AAA) to a stop codon (TAA). This variant was present in 100% of reads from two individuals with CD177^null^ phenotype.

**Table 1:** List of 17 protein-coding variations showed their incidence, genomic location, polymorphisms and alteration in amino acids (A.A.) in GRCh 37 (hg19) and updated version GRCh38 (hg38).

Variations	Prevalence	Region	Position (hg19)	SNV	A.A.	Position (hg38)	SNV	A.A.	Position (*CD177*)	Position (cDNA)
rs45441892	23/39	Exon 1	43,857,873	G->C	A3P	43,353,721	G->C	A3P	g.49	c.9
s45553433	2/39	Exon 2	43,858,044	A->T	H31L	43,353,892	A->T	H31L	g.220	c.92
rs45571738	7/39		43,858,066	G->A	L38L	43,353,914	G->A	L38L	g.242	c.114
novel	39/39	Exon 4	43,859,815	C->G	P128A	43,355,663	C->G	P128A	g.1991	c.381
rs12981714	38/39		43,860,192	T->G	V184G	43,356,040	T->G	V184G	g.2368	c.551
rs12980412	38/39	Exon 5	43,860,251	G->A	D204N	43,356,099	G->A	D204N	g.2427	c.610
rs12981771	38/39		43,860,255	T->G	M205R	43,356,103	T->G	M205R	g.2431	c.614
rs57802244	1/39		43,864,507	T->C	M237T	43,360,355	T->C	M237T	g.6683	c.710
rs10425835	27/39	Exon 6	43,864,548	C->A	L251I	43,360,396	C->A	L251I	g.6724	c.751
novel	39/39		43,865,316	C->G	A261G	43,361,164	G->C	G261A	g.7492	c.782
novel	39/39		43,865,320	C->A	T262T	43,361,168	A->C	T262T	g.7496	c.786
**novel**	**39/39**		**43,865,321**	**T->A**	**X263K**	**43,361,169**	**A->T**	**K263X**	**g.7497**	**c.787**
rs200145410	39/39	Exon 7	43,865,324	A->G	S264G	43,361,172	G->A	G264S	g.7500	c.790
rs200006364	10/39		43,865,325	G->T	S264I	43,361,173	G->T	G264V	g.7501	c.791
rs201266439	39/39		43,865,333	G->A	A267T	43,361,181	A->G	T267A	g.7509	c.798
rs17856829	15/39	Exon 8	43,865,692	G->A	A348T	43,361,540	G->A	A348T	g.7968	c.1022
rs78718189	4/39	Exon 9	43,866,449	G->A	G431R	43,362,297	G->A	G431R	g.8625	c.1281

The incidence of nucleotide variation expressed as a percentage of all reads for each individual is shown in Fig 2B. Read frequencies of 0, 50 or 100% were observed for most SNVs across the cohort, consistent with simple Mendelian inheritance. By contrast, SNVs identified in exons 4, 5, and 7, which were identified in almost all subjects, yielded read frequencies of approximately 25, 50 or 75%, from which we inferred the existence of four alleles (two bi-allelic loci) (Fig 2A and 2B, Table 1, S3A, S3B and S3C Fig). At least two of these loci contained the premature stop codon (g.7497A>T) in all individuals.

We designed a high throughput assay using two-tailed allele specific primers for universal energy-transfer amplification (Amplifluor PCR), which identifies reference g.7497A and variant T alleles [36]. This correctly identified all genotypes defined by deep sequencing, and confirmed that the variant allele occurs with frequencies of 100, 75 and 50% in our test cohort (S3D and S3E Fig). Next, we genotyped the 535 healthy subjects in cohort 2 (S3F Fig) and found a similar distribution of genotypes as in cohort 1 (S3G Fig). Analysis of population frequencies of *CD177* g.7497 genotypes was similar in individuals from each ethnic group (S3H Fig).

### Neutrophil *CD177* variations are germline encoded

SNVs identified by deep sequencing were confirmed by Sanger sequencing (Fig 2C). In addition, we compared sequences obtained from genomic DNA isolated from saliva and neutrophils, to determine whether *CD177* exon 7 variations were transmitted in the germline or arose spontaneously by somatic mutation in neutrophils, and whether variant alleles were represented at different frequencies in individuals with different neutrophil phenotypes. Results were perfectly concordant with those obtained by deep sequencing, and confirmed all *CD177* sequence variants, including the novel stop codon *CD177* g.7497T (K263X), in DNA from both neutrophils and saliva, and consistent with allelic ratios derived from deep sequencing in neutrophil-derived DNA (Fig 2A and 2C).

We also examined all variants by in silico prediction algorithms, as our recent analysis on de novo or low-frequency missense mutations revealed that deleterious effects might be over-estimated in animal models [37]. Although only the stop gain K263X variation segregates with altered CD177 expression, 5 out 17 (35%) coding variations in *CD177* are predicted to be damaging with high scores of PolyPhen2, CADD and SIFT (S2 Table) [38, 39, 40]. A mutation significance cutoff (MSC) study demonstrated that with a 99% confidence interval (CI), *CD177*-specific cutoff for PolyPhen2 and CADD are 0.523 and 5.946 respectively, predicting high impact of five PolyPhen2 predicted and three CADD predicted damaging variations [41].

### *CD177* g.7497A allele frequency correlates with CD177 expression

**Fig 3:**
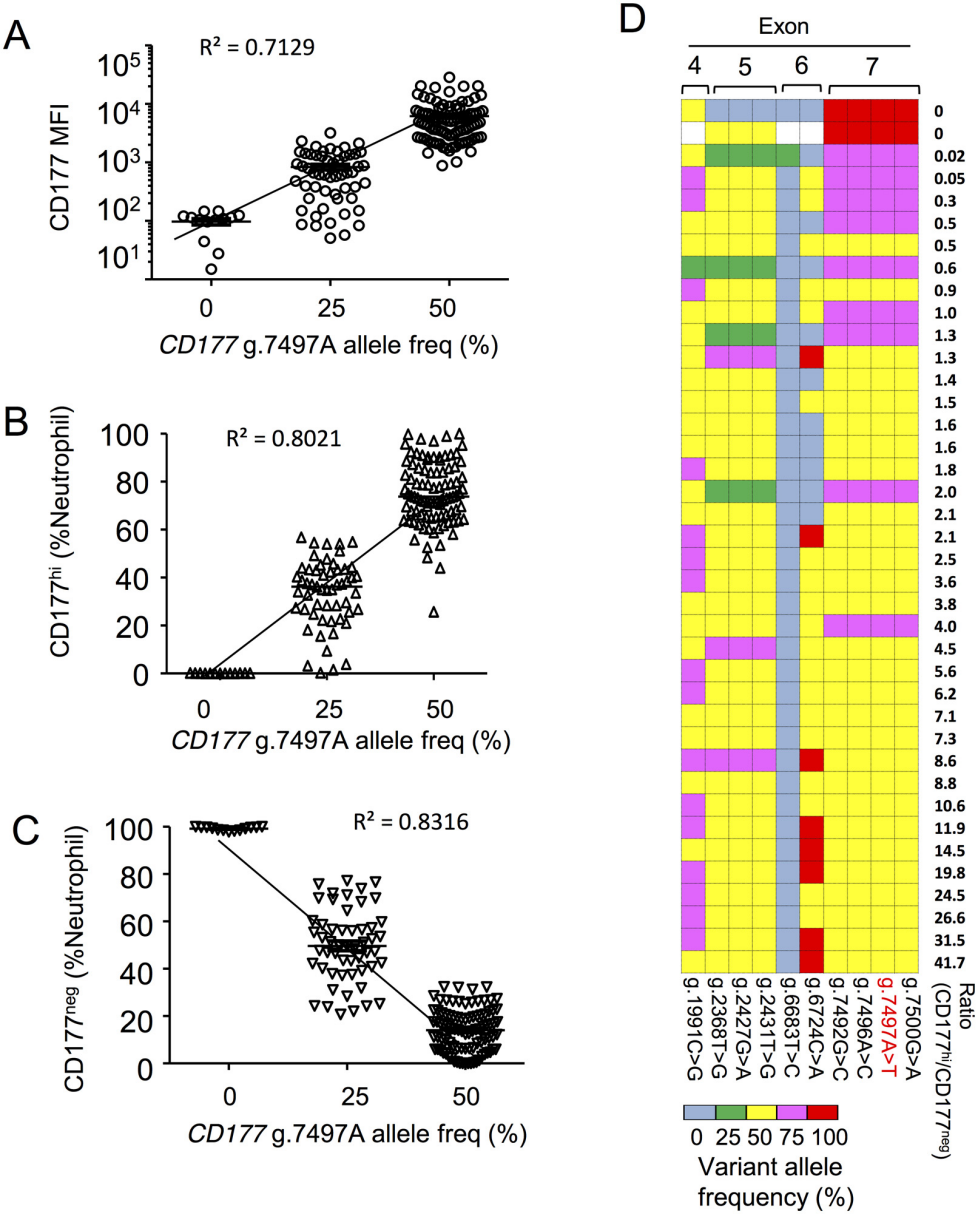
*CD177* g.7497A allele frequencies correlate to neutrophil CD177 expression. **A—C**. Association of reference allele frequency (g.7497A) with neutrophil CD177 expression measured by geometric mean fluorescence intensity (MFI) of CD177 on the cell surface of total neutrophils (**A**), percentages of CD177^hi^ neutrophils (**B**), and CD177^neg^ neutrophils (**C**) in the blood of cohort 2. **D**. Heat map of variant allele frequency (determined by deep sequencing) and neutrophil phenotypes (determined by flow cytometry) in cohort 1.

We examined *CD177* g.7497A allele frequency in individuals with different CD177 phenotypes in both cohorts. This analysis included a total of sixteen CD177^null^ individuals. We observed a strong correlation between expression of CD177 and *CD177* g.7497A allele frequency in cohort 2 (Fig 3A). In particular, all individuals homozygous for *CD177* g.7497T were CD177^null^. Individuals with a *g*.*7497A* allele read frequency of 50% have larger proportions of CD177^hi^ and fewer circulating CD177^neg^ neutrophils than individuals with 25% A alleles (Fig 3B and 3C). These data are consistent with the proposition that presence of the reference g.7497A allele determines neutrophil CD177 expression, whilst the *CD177* g.7497T allele specifies the abundance of CD177^neg^ neutrophils. Exclusive presence of T at g.7497 accounts for the CD177^null^ phenotype.

We expressed genotype-phenotype data according to all nucleotide variants identified by deep sequencing, and by reference to CD177 surface phenotypes in cohort 1 (Fig 3D). This makes obvious the concordance for read frequencies of each exon 7 variant. There is a correlation between each haplotype encompassing exon 7 and CD177 phenotype. We found 100% variant exon 7 reads in all CD177^null^ individuals, 75% variant exon 7 reads in 7/9 of the individuals with the next lowest ratios of CD177^hi^ to CD177^neg^ cells, and 50% variant exon 7 reads in 26/28 individuals expressing the highest proportion of CD177^hi^ cells.

### *CD177* novel variants reflect sequence divergence from *CD177P1* pseudogene

**Fig 4:**
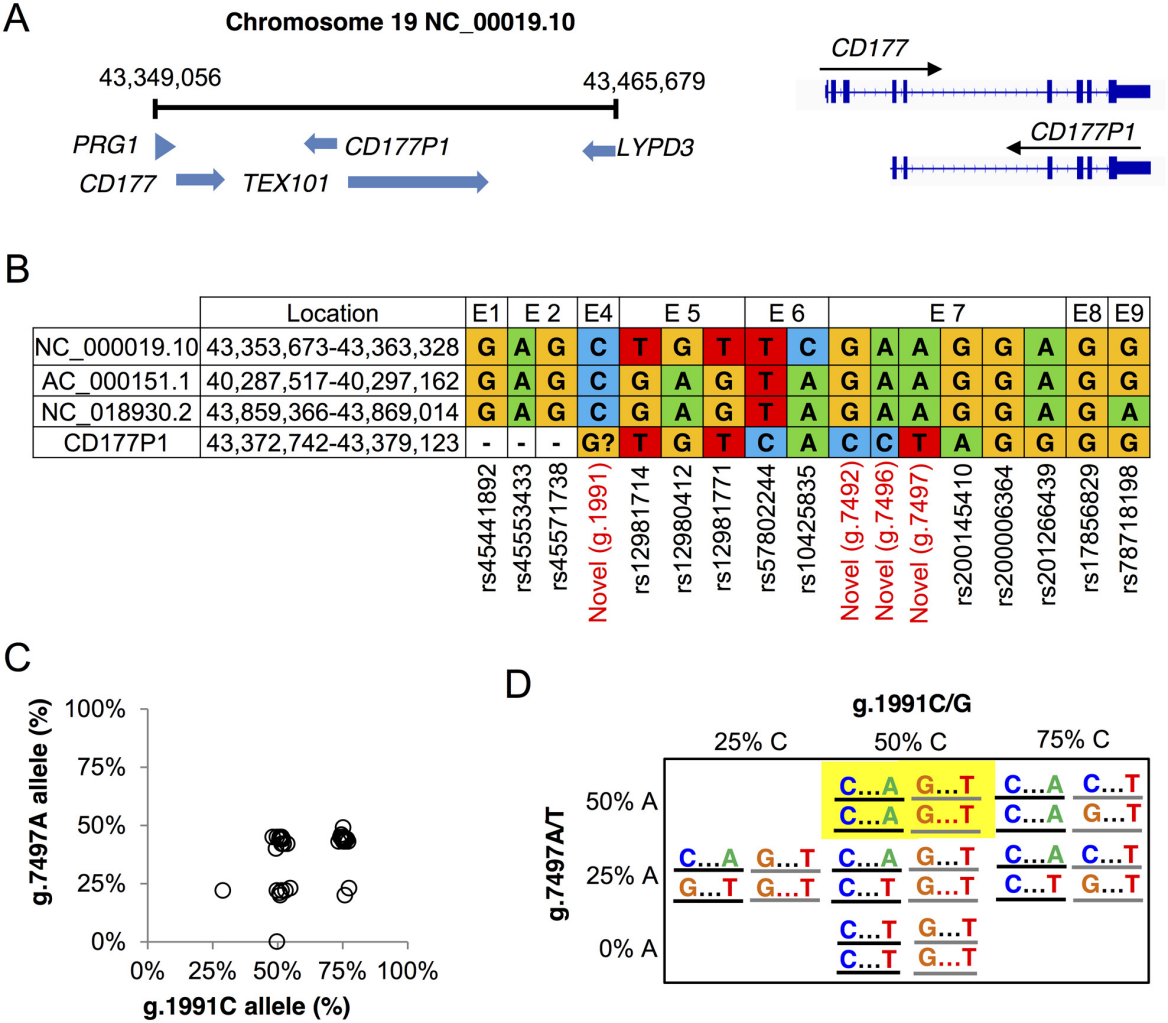
*CD177* and *CD177P1* variations. **A**. *CD177* locus on human chromosome 19 and a schematic comparison of *CD177* and *CD177P1* genes. **B.** Current annotation of three copy number variations of *CD177* and *CD177P1* gene polymorphisms. **C**. *CD177* reference allele frequencies of two polymorphisms, g.1991C in exon 4 and g.7497A in exon 7, of cohort 1. Each dot represents one of 40 tested subjects. 15 out of 40 subjects displayed allele frequencies of g.1991C and g.7497A simultaneously at 50%. 14/40 subjects harboured similarly 50% g.7497A allele but 75% g.1991C. **D**. Proposed *CD177/CD177P1* haplotypes in two loci of exon 4 (C/G) and exon 7 (A/T). *CD177* gene in black line and *CD177P1* in grey. The most frequent genotype is highlighted.

According to Human Genome Assembly 106 (build 38), human chromosome 19 contains *CD177* (CD_00019.10) separated by 10kb from the *CD177P1* pseudogene, which comprises sequences homologous with *CD177* exons 4–9 on the minus strand (NC_000019.10) (Fig 4A). Analysis of these reference sequences, and alternative sequences deposited in GenBank reveals uncertainty over the provenance of the variants we identified (Fig 4B). Comparison of human reference *CD177* gene sequences with those from other mammalian species is informative for resolving this uncertainty (S4A, S4B and S4C Fig). The g.1991C>G variant identified in exon 4 (43,355,663, c.381C>G), which causes a proline to alanine (P128A) substitution, has not been reported in any of the reference sequences, and is not annotated in dbSNP. The exon 5 variants we identified as heterozygous in 39/40 subjects appear as discrepancies in reference sequences and probably reflect differences between *CD177* gene and *CD177P1*. Most significantly, the exon 7 variant haplotype containing g.7497T appears to arise from *CD177P1* (Fig 4B). We postulated that the CD177^null^ phenotype arises when exon 7 reads are derived exclusively from *CD177P1*, while *CD177* exon7 sequence is not detected in the genome of these individuals.

The sequence homolog between *CD177* and *CD177P1*, along with detection of two bi-alleles of *CD177* exon 4, 5 and 7 as described above, suggested that variations in these regions may reflect sequence divergence from *CD177P1*. To explore this proposition further, we examined the relation between variation g.1991C at 43,355,663 locus in exon 4 and g.7497A (43,361,169) in exon 7 across cohort 1 (Fig 4C and 4D). We recorded read frequencies of 25, 50 and 75% for g.1991C, but read frequencies of 0, 25 and 50% for g.7497A. Our observation of a maximum of 50% g.7497A reads is consistent with homozygosity for the T allele at the *CD177P1* locus in the population. This was also most frequently observed in 29/40 subjects, in which 15/40 subjects were 50:50 heterozygous at both g.1991 in exon 4 and g.7497 in exon 7, suggesting homozygous reference alleles of C/C and A/A in the two loci of *CD177* gene and ‘variant’ alleles G/G and T/T in *CD177P1*. Other possibilities included homozygosity at one locus but heterozygosity at the other, or heterozygosity at both loci. Possible haplotypes are shown in Fig 4D. Analysis of haplotypes between exon 4 and exon 5 showed similar results. Interestingly, linkage disequilibrium (LD) analysis with Genome1000 data are consistent with a haplotype block encompassing *CD177P1* and only the 3’ region of *CD177* (S5 Fig).

Phylogenetic analysis reveals *CD177*-like sequence in orang-utan and pygmy chimpanzee (S6 Fig). However, the exonic structure of CD177 varies considerably between mammalian species (S7 Fig), with evidence of gene duplication giving rise either to *CD177*-like genes (S8 Fig) or to CD177 itself. Thus, mouse *Cd177* comprises 17 exons, and with significant nucleotide and protein sequence homology between the first and second halves of the molecule (S9 Fig). Both halves of mouse CD177 exhibit approximately 50% amino acid homology with human CD177 (S10 Fig).

### CD177 expression is a heritable trait determined by the ratio of *CD177/CD177P1* alleles

**Fig 5:**
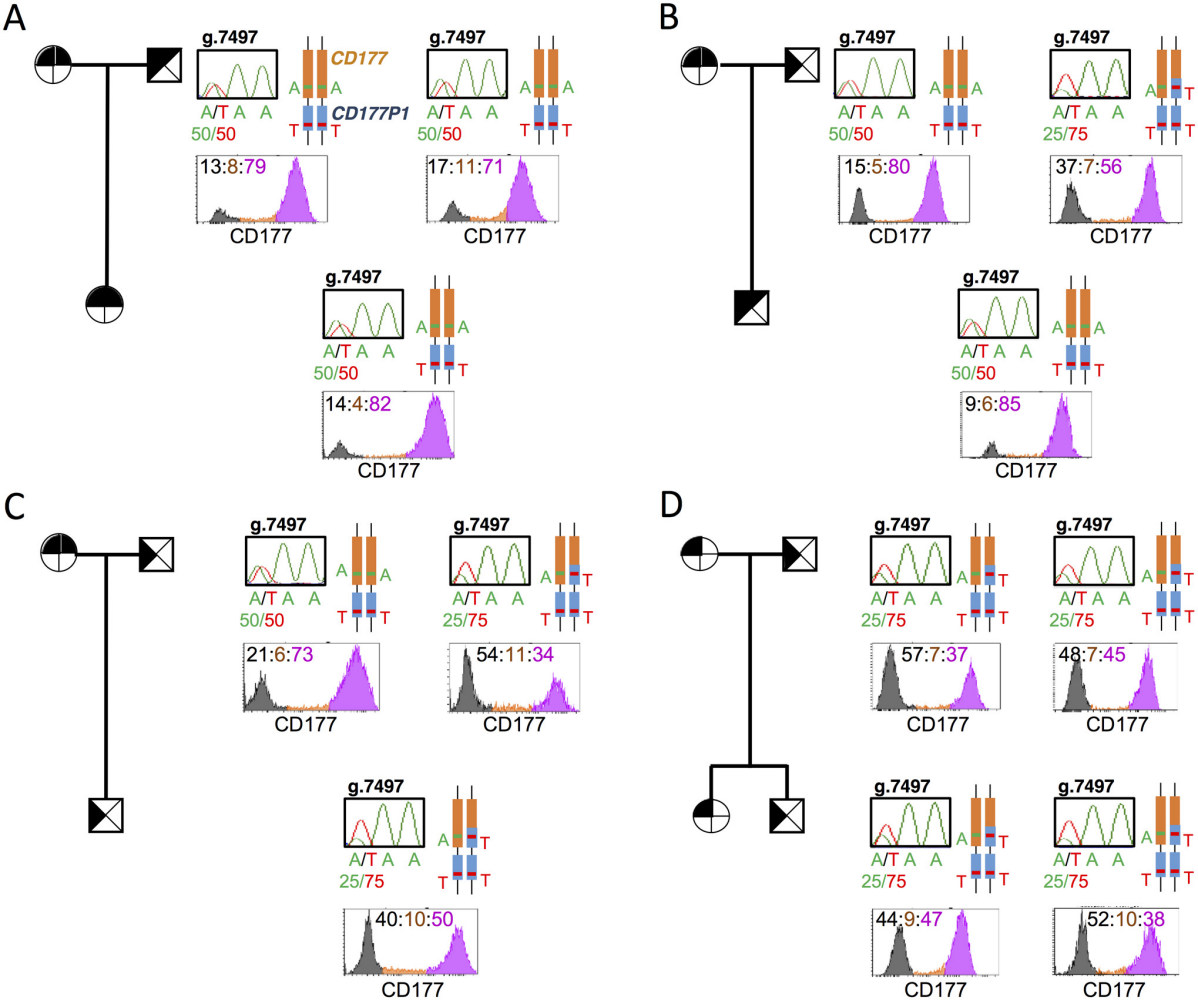
An inheritable phenotype of CD177 determined by ratio of *CD177/CD177P1* alleles. **A-D**. Pedigrees of 4 unrelated families with different CD177 geno- and phenotypes, all exhibit Mendelian inheritance. ⊕: female, ⊠: male; filled: *CD177* exon 7, blank: *CD177P1* exon 7. Genotypes were also shown in schematic structures, *CD177* in orange and *CD177P1* in blue, with g.7497A/T polymorphisms labelled in both alleles at *CD177* and *CD177P1* loci. Chromatograms showed genomic sequence trace of *CD177* g.7497 labelled with ratio of Ref:Var in each subject. Histograms showed CD177 phenotypes, numbers in corresponding colours indicating percentages of neutrophil subsets, CD177^neg^ in black, CD177^int^ in orange, CD177^hi^ in pink.

As another approach to evaluate the association between *CD177* and *CD177P1* genotypes and CD177 phenotypes, we examined CD177 expression according to the genotypes of parents and their offspring in families where parents exhibit different ratios of A and T at *CD177*.g7497 (Fig 5A–5D). In pedigree 1, both parents exhibit CD177^hi^ phenotypes, and are sequenced for *CD177*.g7497A/T at 50:50 ratio according to electropherogram, from which we infer homozygous g.7497A in *CD177*, since *CD177P1* is homozygous T. Consistent with this, their offspring shares the same genotype and phenotype (Fig 5A). In pedigree 2, the maternal phenotype is CD177^hi^ and 50:50 *CD177*.g7497A/T, while the paternal phenotype is CD177^hi/neg^, with 25:75 *CD177*.g7497A/T, from which we infer A/T heterozygosity for *CD177*. The offspring is genotyped as 50:50 g.7497A/T (inheriting a reference *CD177* allele from each parent), and exhibits a CD177^hi^ phenotype (Fig 5B). By contrast, pedigree 3 illustrates similar parental genotypes and phenotypes to pedigree 2, but the offspring is 25:75 *CD177*.g7497A/T according to the electropherogram and exhibits a CD177^hi/neg^ phenotype, suggesting a *CD177* variant allele from the father (Fig 5C). Finally, in pedigree 4, both parents have 25:75 g.7497A/T genotype and CD177^hi/neg^ phenotypes, and both offspring inherit similar phenotypes and genotypes (Fig 5D).

### *CD177/CD177P1* divergence confirmed by comparison of gDNA and cDNA sequences

**Fig 6:**
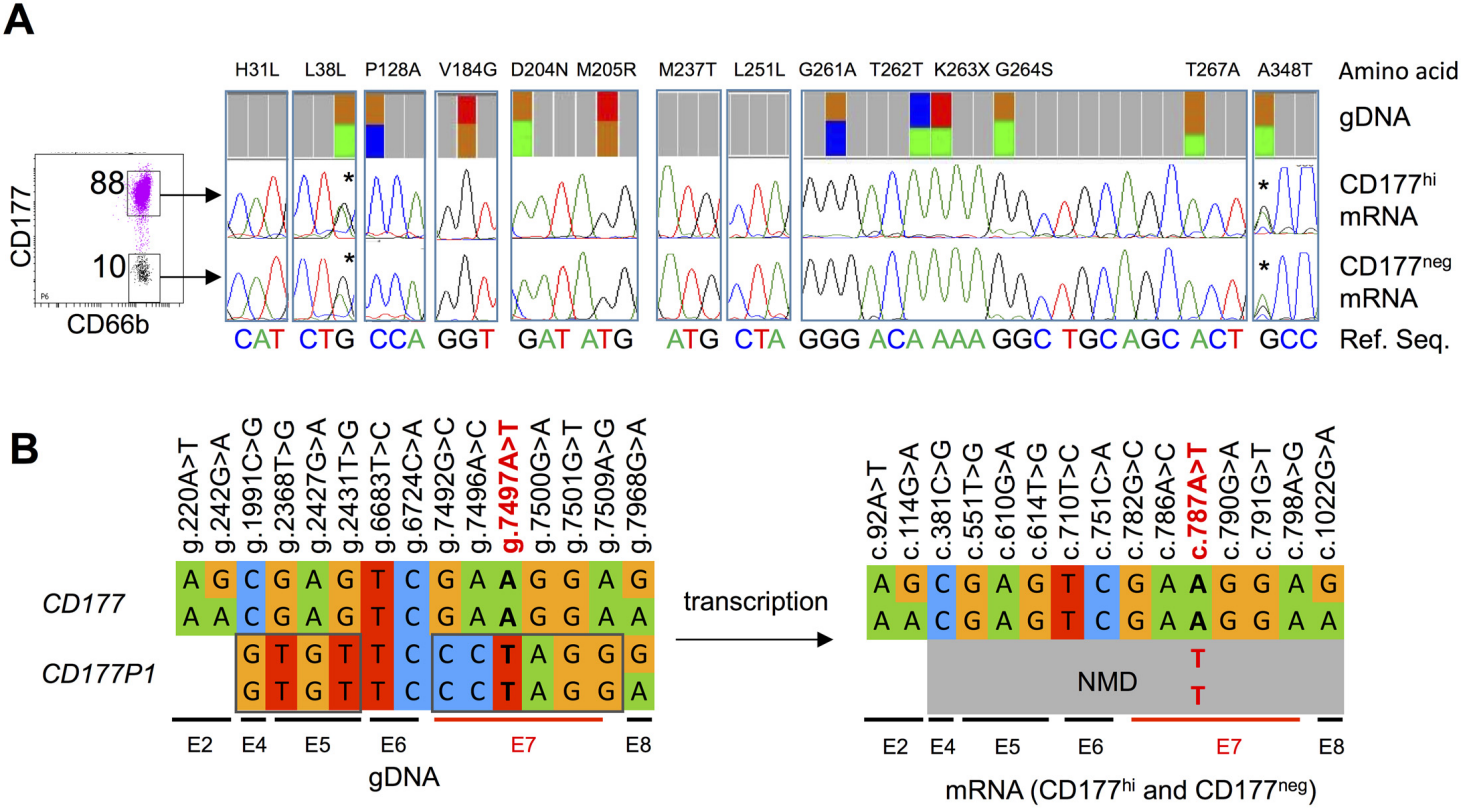
Analysis of *CD177* variations and *CD177P1* divergence from gDNA and cDNA sequence. **A**. CD177^neg^ and CD177^hi^ cells were sorted from an individual who predominantly express CD177^hi^ in neutrophils (*left*). Sequencing traces showed *CD177* codon variations at indicated loci, comparing results from gDNA deep sequencing (top), CD177 mRNA isolated from purified CD177^hi^ cells (middle) and CD177^neg^ neutrophils (bottom). The reference nucleotide sequences were labelled in colour letters below. The two SNPs present in gDNA and cDNA of both cell subsets were labelled (*). **B**. A schematic summary of the variations in gDNA and mRNA in neutrophils of this subject. Sequence variations were indicated on the top of the gene/transcript and exons were indicated (E2, E4 etc) below. *CD177P1* nucleotides in exon 4, 5 and 7 were outlined. CD177P1 transcripts were shown in grey signifying expected NMD with the stop codon variation g.7497T in red.

In order to resolve the uncertainty of *CD177* reference sequence, we compared sequences of *CD177* gDNA and mRNA isolated from individuals who exhibited different CD177 phenotypes and harboured putative *CD177* polymorphisms. We sorted CD177^hi^ and CD177^neg^ subsets from a subject whose 88% of neutrophils in the blood were CD177^hi^, amplified full-length CD177 cDNA from both subsets, and compared them with genomic DNA sequences determined by deep sequencing (Fig 6A). In individuals whose neutrophils are predominantly CD177^hi^, CD177 transcripts are homozygous c.787A. Variant c.787T transcripts were not detected, consistent with prediction of nonsense mediated decay (NMD) of CD177P1 transcripts.

We identified 11 *CD177* polymorphisms in exons 2, 4, 5, 7 and 8, all apparently heterozygous (approximately 50% of reads). Exon 2 is absent from *CD177P1*, therefore, we inferred that g.242G>A is a SNP in *CD177*, which was confirmed by analysis of cDNA sequences (Fig 6B). Similarly, g.7968G>A in exon 8 appears to be a *CD177* SNP, whereas other putative polymorphisms in exons 4, 5 and 7 were not found in CD177 transcripts (Fig 6A), suggesting that they represent divergence between *CD177* and *CD177P1* rather than *CD177* SNPs (Fig 6B). In summary, g.1991C (exon 4), g.2368G, g.2427A and g.2431G (exon 5), and g.7492G, g.7496A, g.7497A, g.7500G, and g.7509A (exon 7) are *CD177* gene reference sequences, and variants at these loci are actually derived from *CD177P1*.

### Incorporation of *CD177P1* exon 7 into *CD177* locus causes distinctive CD177 transcription in CD177^neg^ neutrophils

**Fig 7:**
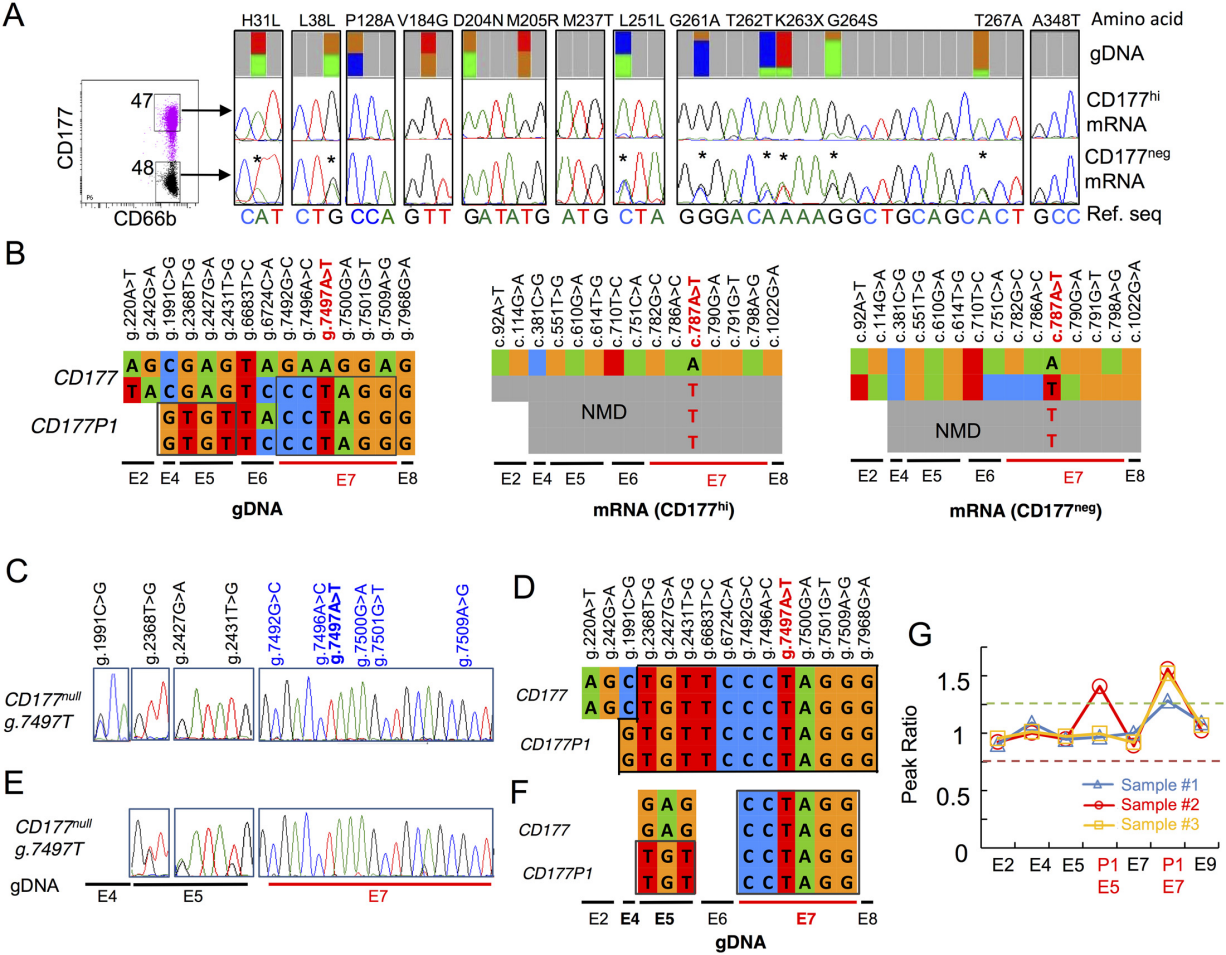
Ectopic and allelic *CD177P1* exon 7 conversion. **A**. CD177^hi^ and CD177^neg^ neutrophils were sorted from a single donor with bimodal CD177 expression (*left*). Genomic variant allele frequencies were determined by deep sequencing (*top*), and compared with sequence variations in cDNA from CD177^hi^ (*middle*) and CD177^neg^ neutrophils (*bottom*). Two *CD177* transcripts were found in CD177^neg^ neutrophils. SNPs present in gDNA and cDNA of CD177^neg^ subsets but absent from CD177^hi^ cells are labelled (*). **B**. Schematic summaries of *CD177* vs *CD177P1* gDNA variations and CD177 mRNA in two neutrophil subsets. *CD177P1* derived nucleotides in exon 4, 5 and 7 are outlined, suggesting one *CD177* allele partially supplied by *CD177P1* exon 7. **C**&**E**. Genomic sequence traces in indicated loci within exon 4, 5 and 7 of two CD177^null^ individuals. Only *CD177P1* exon 7 sequences are detected in CD177^null^ subjects, who harbour both *CD177* and *CD177P1* upstream elements, i.e., exon 4 (**C**) and exon 5 (**E**). **D**&**F**. Schematic genomic *CD177*/*CD177P1* structures of CD177^null^ individuals as shown in **C**&**E**. **G**. Confirmation of ectopic and allelic *CD177P1* exon 7 conversion in three subjects by MLPA. The plots show the peak ratio of probes for indicated loci of *CD177* and *CD177P1* genes. Exon 2 is used as a reference read out of the *CD177* gene only; probes for exon 4, 5, 7 and 9 bind to both *CD177* and *CD177P1*, whereas probes labelled as (P1) are specific to *CD177P1* exon 5 and 7. The graph shows one copy duplication of *CD177P1* exon 7 in blue in the same subject as shown in **A** & **B**; and two copies duplication (allelic conversion) in the two CD177^null^ subjects (red & orange) as shown in **C-F**. The subject in red also shows duplication of *CD177P1* exon 5 in concordant to **C** & **D**.

Next, we investigated the *CD177* sequences in individuals with 25:75 ratio of g.7497A/T genotype, which confers CD177^hi/neg^ neutrophil phenotypes. Once again, we compared genomic and transcript sequences but this time from a subject with approximately equal distributions of CD177^hi^ and CD177^neg^ neutrophils in peripheral blood. gDNA sequences revealed similar abundance of variations and references bases for seven SNPs in exons 2, 4, 5 and 6. By contrast, we observed a 25:75 ratio (reference to variant allele) for five SNPs in exon 7 (Fig 7A), consistent with presence of one copy of *CD177* exon 7 and three copies of *CD177P1* pseudogene derived sequence. A possible explanation is that one *CD177* allele was partially replaced with *CD177P1* homolog via ectopic gene conversion, yielding a chimeric *CD177* allele containing a pre-mature stop codon (g.7497T) in exon 7 (Fig 7A and 7B).

This hypothesis was lent additional support by the presence of distinctive CD177 transcripts in CD177^neg^ and CD177^hi^ cells within the same individual (Fig 7A). Monomorphic CD177 mRNA transcripts from the reference *CD177* allele were recovered from CD177^hi^ neutrophils. By contrast, two different transcripts were recovered from CD177^neg^ neutrophils. Besides a same copy of CD177 reference transcript, a variant transcript was also recovered containing eight SNPs: c.92A/T, c.114G/A, c.751C/A, c.782G/C, c.786A/C, c.787A/T, c.790G/A and c.798A/G in CD177^neg^ neutrophils. These SNPs corresponded exactly to genomic heterozygosity of g.220A/T and g.242G/A (exons 2), g.6724C/A (exon 6) and g.7492G/C, g.7496A/C, g.7497A/T, g.7500G/A and g.7509A/G (exon 7). Again, SNPs identified in exon 2 (g.220A>T and g.242G>A) and exon 6 (g.6724C>A) represented common variations in *CD177* gene, whereas SNPs in exon 7 arose from *CD177P1*. *CD177P1* derived exon 7 sequences were recovered in the cDNA. This demonstrated the expression of chimeric CD177 transcripts and supported the proposition of *CD177P1* exon 7 incorporation in *CD177* locus (Fig 7A and 7B). This finding proved that both intact and converted *CD177* alleles are transcribed in CD177^neg^ neutrophils, whereas CD177^hi^ neutrophils express only reference allele. A mechanism of ectopic gene conversion also explains 25:75 (ref/var) ratio of polymorphisms in exon 7 of the gene.

To confirm this structural change, we performed MLPA using probes specific to different regions of *CD177* and *CD177P1* genes in relation to individuals with normal copy numbers of both genes. This analysis confirmed the presence of an additional copy of *CD177P1* exon 7 (Fig 7G, sample 1 in blue), in concordance with deep sequencing data (Fig 7A). These results indicate ectopic gene conversion of *CD177P1* exon 7 into the *CD177* locus, resulting in 25:75 ratio of *CD177* g.7497 A/T alleles.

### Allelic gene conversion of *CD177P1* results in CD177^null^ phenotype

All CD177^null^ subjects were homozygous for *CD177P1* derived exon 7 sequence, suggesting an allelic gene conversion in the region (Figs 2A–2C, 7C–7F, S2D and S2F). Analysis of upstream variations implied different homologous recombination events among CD177^null^ individuals. One subject harboured g.1991C>G polymorphism at 50:50 ratio (Fig 7C), indicating co-existence of two alleles of *CD177* exon 4 (homozygous g.1991C) and two alleles of *CD177P1* (homozygous G). By contrast, only *CD177P1* derived sequence was found from exon 5 to exon 7 in the same individual, suggesting replacement of *CD177* exon 5 to 7 by *CD177P1* homolog in both alleles, and chromosomal crossover occurred between exon 4 and 5 (Fig 7C and 7D). Similarly, presence of both *CD177* and *CD177P1* sequences in exon 5 but exclusive *CD177P1* sequence in exon 7 in another CD177^null^ subject indicated homologous recombination between exon 5 and 7 (Fig 7E and 7F). MLPA confirmed the presence of 4 copies of *CD177P1* exon 7 in both CD177^null^ subjects, demonstrating allelic *CD177* gene conversion (Fig 7G). Furthermore, duplication of *CD177P1* exon 5 in one subject (red) but not in another (orange) confirmed various homologous recombination occurred in CD177^null^ subjects (Fig 7G).

**Fig 8:**
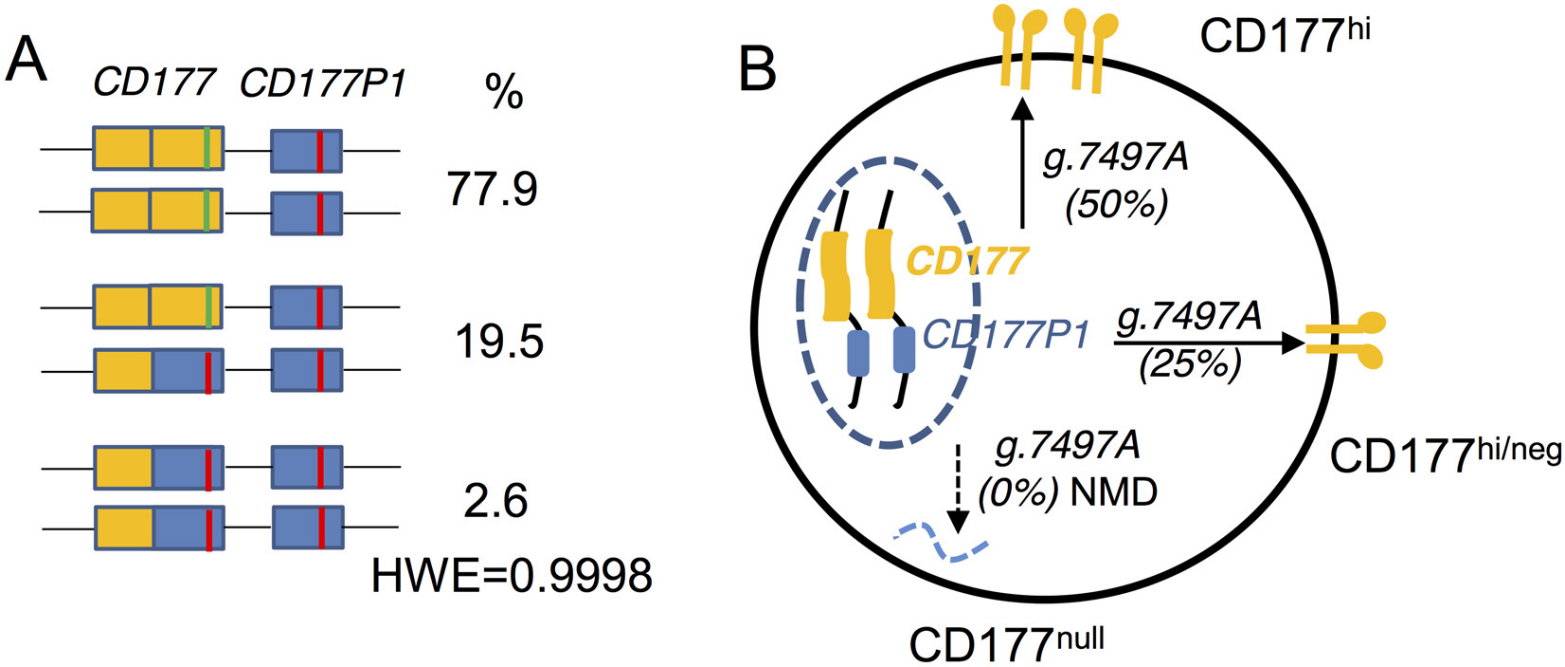
Models for *CD177* allelic arrangement and genotype-phenotype relation. **A**. A proposed allelic arrangement model to account for *CD177* variant read frequencies. *CD177P1*-derived segments are shown in blue boxes and g.7497T in red lines. *CD177* genes are in yellow and g.7497A in green. Prevalence of each arrangement in cohort 2 is shown, together with calculated Hardy-Weinberg ratio. **B**. Model to account for observed CD177 genotype-phenotype relation.

Our data from both deep sequencing and MLPA support an allelic gene conversion by *CD177P1* exon 7 in CD177^null^ subjects. *CD177* exon 7 had been mistakenly annotated as a polymorphic pseudogene in GRCh37. It should be noted that current understanding for exon 5 sequence was incorrect too. Our data suggested that “reference” g.2368T, g.2427G and g.2431T according database were actually linked with other *CD177P1* elements and “variant” genotypes of g.2368G, g.2427A and g.2431G should be annotated as *CD177* sequence (Figs 6 and 7). Complete *CD177* sequence in alignment with *CD177P1* is shown in S11 Fig Taken together, these findings indicate that the stop codon responsible for the CD177^null^ phenotype is derived from *CD177P1*. This chimeric *CD177* gene has arisen by gene conversion. This would be consistent with both the allelic frequencies observed for exon 7 haplotypes, with the transcriptional analysis, and with gene structural analysis by MLPA. The allelic frequencies are in Hardy-Weinberg equilibrium (= 0.9998) based on the genotype frequencies in our large cohort 2 (Fig 8A and 8B).

## Discussion

We report evidence for the genetic specification of heterogeneous CD177 phenotypes by ectopic and allelic conversion. We identified a novel polymorphism in exon 7 (g.7497A>T), encoding a stop codon in place of a lysine codon. Based on analysis of more than 9000 nucleotide reads from each individual and a large cohort over 500 subjects, we determined with a high level of confidence that this codon is present in all individuals. Most individuals have both lysine and stop codons detected, whereas in CD177^null^ individuals, only the stop codon is detected which is derived from *CD177P1* exon 7 conversion.

Previous studies have identified variations in the transcript of CD177 deficient individuals with no satisfactory explanation for the origin of the splicing error [30, 31]. Other investigators have identified SNVs within *CD177* gene in association with expression [32, 33], and although this resulted in some progress, were ultimately inconclusive either because of the absence of full length genomic sequence of *CD177*, or the absence of CD177^null^ subjects (S1 Table). As a result, the mechanisms that account or the CD177^null^ phenotype have not been resolved. Recently, Li and colleagues independently identified the same putative nonsense mutation in *CD177* gene reported here, and demonstrated that this variation account for lack of CD177 expression in transfected cells [34]. Our finding that the null allele arises by conversion from a pseudogene with close sequence homology helps to explain why previous results have been inconclusive. Indeed, we show that the *CD177* reference sequences lodged in GenBank contain inconsistencies, and have resulted from assembly of sequences from the gene and pseudogene.

*CD177P1* comprises orthologs of exons 4–9 of *CD177* gene. Previous studies have unsuccessfully attempted to resolve the contributions of the *CD177P1* [42, 43]. Resolution of this uncertainty has been achieved here with deep sequencing of captured *CD177* alleles. We have identified portions of the *CD177* gene that harbour variants with strict Mendelian inheritance, whereas exon 7 exhibits variant frequencies that could not be accounted for by the presence of just two alleles.

Our findings demonstrated that *CD177* is sometimes a chimeric gene resulting from incorporation of a gene segment derived from *CD177P1* (including exon 7). We provide evidence in this study that this chimeric gene has arisen by gene conversion. Gene conversion is a process of homologous recombination involving unidirectional transfer of genetic material to duplicated gene from its ancestor such as pseudogene [44]. A similar mechanism of gene conversion from pseudogenes resulting in insertion of a nonsense codon has been reported in chronic granulomatous disease, polycystic kidney disease, and B cell immune deficiency [45–47]. The presence of the homologous sequences in *CD177* and *CD177P1* has resulted in misannotation of *CD177*, and thwarted efforts to identify the genetic basis for the CD177^null^ phenotype.

The explanation we propose for CD177^null^ alleles within the population also appears to account for phenotypic heterogeneity. We observed a marked concordance between levels of CD177 expression and the number of *CD177* exon 7 alleles. Thus, one allele (g.7497A read frequency of 25%, by ectopic *CD177P1* conversion) is associated with lower levels of CD177 expression than two alleles (g.7497 read frequency 50%). Allelic frequency is supported by analysis of transcripts within neutrophils of subjects having various CD177 phenotypes.

CD177 expression not only varies across the population, but also within individuals. CD177^neg^ cells appear to be distinguished from CD177^hi^ neutrophils. In individuals heterozygous for *CD177* exon 7, CD177^neg^ cells harbor two CD177 transcripts containing *CD177P1*-derived exon 7 sequences, whereas CD177^hi^ cells express only *CD177*-exon 7 containing transcripts. We have identified allelic and ectopic gene conversion as driving forces for CD177^null^ and CD177^neg^ expression respectively. Additional investigations are merited to explore mechanism of atypical CD177 expression (i.e., CD177^int^ subset) within individuals, which might include epigenetic changes and posttranscriptional regulation.

Absence of self-antigen predispose to a breakdown in immunological tolerance upon exposure to self-antigen. Failure to acquire self-tolerance to neutrophil antigens places an individual at risk of developing antibodies to these antigens, either as natural antibodies, or after immunisation. For neutrophil antigens, this is likely to occur after exposure to fetal antigens, or less likely, after blood transfusion or allotransplantation. The consequences of passive transfer of neutrophil antibodies include TRALI, and neonatal immune neutropenia. Indeed, the first description of neonatal alloimmune neutropenia arose in the offspring of CD177^nul^ mothers [11]. Despite the pathogenic role for anti-CD177 antibodies in TRALI and NAN, the lack of a method to genotype *CD177* has prevented CD177 deficiency from being investigated in TRALI causing donors, TRALI patients [48], and pregnant women. For the first time, our study established a method to genotype CD177^nul^ individuals with risk of anti-CD177 antibody development after pregnancies and infusions. Prospective studies will be necessary to characterise in more detail the requirements for alloimmunisation and antibody production in CD177^null^ individuals.

In summary, we have identified the genetic variant that accounts for the CD177^null^ phenotype and heterogeneous CD177 expression. The mechanism appears to result from insertion of a pseudogene derived sequence into the *CD177* locus. Nevertheless, this event can be identified as apparent homozygosity for g.7497 of *CD177* gene. This discovery makes it possible to screen individuals at risk of CD177 isoimmunisation.

## Methods

### Study subjects

Study subjects consisted of 40 patients with anti-neutrophil cytoplasmic autoantibodies (ANCA) associated vasculitis (AAV) (cohort 1) and 535 healthy subjects (cohort 2) were examined for CD177 genotypes and phenotypes, in an effort to elucidate neutrophil mediated autoimmunity. All research described was approved by the ACT Health Human Research Ethics Committee, under protocols ETH.11.11.269 and ETH.1.15.15. Participating subjects provided written informed consent.

### Neutrophil isolation, flow cytometry and cell sorting

Anticoagulant citrate dextrose solution-treated fresh blood was layered on Ficoll-Paque Plus separation medium (GE Healthcare Life Science) at room temperature for 45 minutes to allow erythrocytes to sediment. Leukocytes with minimal residue erythrocytes after sedimentation were carefully layered on the top of 10 ml Ficoll-Paque Plus separation medium and centrifuged at 400g for 40 minutes without brake at room temperature. Neutrophils and peripheral blood mononuclear cells (PBMC) were then recovered to separate tubes [49].

Leukocytes (approximately 1x10^6^) were stained with fluorescent conjugated antibodies for CD16 (3G8), CD66b (G10F5) from Biolegend, CD177 (MEM-166) from Abcam and CD177 (REA258) from Miltenyl Biotec in Ca^++^Mg^++^ free HBSS. Data were acquired on a FACSCanto II flow cytometer (BD Bioscience) and analysed using FlowJo software (TriStar). 1–2 million of CD177^neg^ and CD177^hi^ subsets were sorted on a BD FACS Aria II from CD66b^+^ neutrophils for sequencing and RNA analysis.

### Sanger sequencing, amplifluor PCR, RT-PCR and MLPA

Genomic DNA was extracted from neutrophils and saliva using DNeasy Blood kit (Qiagen) and Oragen-DNA OG-500 kits (DNAgenoTec) respectively. CD177 exon 5 and 7 sequence was amplified with primers (CD177E5F: CAGCATCACTGACTCTCCC TC; CD177E5R: ATGCCCCATGTGTCATCGTG; CD177E7F: AGCTTTCCCTCTCACCCTC AG; CD177E7R: TCTGGGCCTCATTTCTCCACG), and examined in the Bioscience Research Facility.

Two allele specific forward primers and a single common reverse primer were designed to amplify across the polymorphisms at 19:43,361,164 (GRCh38) (*CD177*. g7492G/C). GAAGGTGACCAAGTTCATGCTGACTCACATCAACCCTGGTGGG (CD177F-1) and CD177F-2 (GAAGGTCGGAGTCAACGGATTGACTCACATCAACCCTGGTGGC) both had a 5’ tail corresponding to fluorophores FAM and HEX respectively. The common reverse primer was 86bp downstream within exon 7(CD177-R: CGAGGAGCAGAAGTGGGTAT). Amplification cocktails were prepared with KASP Master Mix (LGC Group). Fluorescences were measured after amplification. Allelic frequencies were discriminated using FLUOstar OPTIMA (BMG Labtech).

Neutrophil RNAs were extracted with TRIzol reagent (Invitrogen) and reverse transcribed into cDNA using Qiagen Reverse Transcription kit. Full length of CD177 transcript was amplified and sequenced with a pair of primers (CD177F: CTGGGGTTCATCCTCCCACT; CD177R: TTAGCAGGAAGGGCAAACCA).

Multiplex ligation-dependent probe amplification (MLPA) were performed using the MRC-Holland Salsa MLPA EK1 FAM reagent kit [50]. Probes were designed based on either homology or discrepancy between *CD177* and *CD177P1* genes following previously described criteria [51]. Oligonucleotides from Sigma-Aldrich are listed in Table 2. Probe mixes were prepared in water with each oligonucleotide at a final concentration of 4 fmol/ul and MLPAs were performed using 100ng gDNA. The products were separated by an ABI 3730 DNA Analyzer (Applied Biosystems). Trace data were analyzed using GeneMarker (Softgenetics). Peak heights were normalized to the average peak height of the control probes followed by normalization to the average peak height of the control samples in cohort 1 whose sequences indicated to have one copy of *CD177* and *CD177P1* gene respectively per chromosome. Threshold values for deletion were set at 0.75 and 1.25 for duplication.

**Table 2:** MLPA probes.

Probes	Oligos	Primer Size (bp)	Probe Length (bp)	Target sequence	homolog
Exon 2_L	GGGTTCCCTAAGGGTTGGACCTGACTCATTCATCCATTAGACTTGGGGTGCCAGGACACGTTGATG	47	124	43353956–43354017	100%
Exon 2_R	[Phos]CTCATTGAGAGCGGTGAGAAGGCCCTGGCGTGCAGAGTCTAGATTGGATCTTGCTGGC	37			
Exon 4_L	GGGTTCCCTAAGGGTTGGACAGGATCCTTGAGGTGCCCAGTCTGCTTGTCTATG	35	106	43355664–43355729	100%
Exon 4_R	[Phos]GAAGGCTGTCTGGAGGGGACAACAGAAGAGATCTAGATTGGATCTTGCTGGC	31		43379055–43379120	100%
Exon 5_L	GGGTTCCCTAAGGGTTGGAGGTGGTCCTGGAGGCAGCATCACTGACTCTCCCTCGCTCCCCCTTT	46	119	43355942–43356018	100%
Exon 5_R	[Phos]CTGCAGGAGGCATCTTCTCCAATCTGAGAGTCCTCTAGATTGGATCTTGCTGGC	33		43378765–43378841	100%
Exon 5 (P1)_L	GGGTTCCCTAAGGGTTGGATCTATGACTAGGGGCAGGACTCACCTTTCATATCG	35	112	43356066–43356125	96%
Exon 5 (P1)_R	[Phos]CAGTTCTCAGTCATACCCACGGGCCCAATTTCAGATCTCTAGATTGGATCTTGCTGGC	37		43378658–43378717	100%
Exon 7_L	GGGTTCCCTAAGGGTTGGAGTGCTTGTGGCCTCCTATACCCACT	25	89	43361235–43361283	100%
Exon 7_R	[Phos]TCTGCTCCTCGGACCTGTGCAATATCTAGATTGGATCTTGCTGGC	24		43373497–43373545	100%
Exon 7 (P1)_L	GGGTTCCCTAAGGGTTGGACTGGTGGCGACCTAAAGCTGCAGC	24	87	43361173–43361203	89%
Exon 7 (P1)_R	[Phos]GCTGTTGGGGCTCAAAATTCCCATCTAGATTGGATCTTGCTGGC	23		43373577–43373623	100%
Exon 9_L	GGGTTCCCTAAGGGTTGGACCTGGACTCCTGGGTTTATGAATTTG	26	97	43362013 43362072	100%
Exon 9_R	[Phos]GCTGGGCTGTACTCTGTGTCCTTTCTGACTTTCTAGATTGGATCTTGCTGGC	32		43372990 43373050	100%
Control probes					
C1_chr22_L	GGGTTCCCTAAGGGTTGGAGCCAACCTAAGCACTGTTAGTCAGATTGATCCCAGCTCCAT	60	117		
C1_chr22_R	[Phos]AGAAAGAGCCTATGCAGCTCTTGGACTACCCTATCATCTAGATTGGATCTTGCTGGC	57			
C2_chr9_L	GGGTTCCCTAAGGGTTGGACCTTGAATGTGAGCCTGCGTCGGAGCCCAGCAGCTTCACAGTCACTCCCGT	70	135		
C2_chr9_R	[Phos]CATCGAGGAGGACGAGTGAGCAGTGCCTGCTGCCGATGGCGGTTTCTAGATTGGATCTTGCTGGC	65			
C3_chr17_L	GGGTTCCCTAAGGGTTGGAGTAACTGGAATTCACTCACAGAACATGCTG	49	109		
C3_chr17_R	[Phos]CACTCTTCCTCAACTCAAACTGAGTATCCAGtatcggatTCTAGATTGGATCTTGCTGGC	60			

### Custom exon capture, deep sequencing, and data analysis

TruSeq Custom Amplicon libraries were prepared according to the manufacturer’s instruction (Illumina). 76 pairs of primers were designed for amplicons, covering the entire coding regions of three neutrophil antigen genes including *CD177*. High through-put paired-end sequencing was performed on the Illuminia MiSeq platform for 500 cycles in the Bioscience Research Facility. Primary processed FATSO files were analysed with MiSeq reporter and a homemade pipeline developed by the Immunogenomics Bioinformatics team. BAM files were viewed with integrative genomics viewer (the Broad Institute), comparing to reference human genome hg19 and converted to GRCh38 (hg38).

## Supporting Information

S1 Table Summary of studies on CD177 variations and expression. (PDF)

S2 Table In silico analysis of CD177 single nucleotide variants. (PDF)

S1 Fig A schematic structure of CD177 transcripts with premature stop codons identified. (PDF)

S2 Fig A. Ethnicity of the study subjects in the two cohorts. B. Prevalence of CD177 phenotypes in both cohorts. C-E. Analysis of neutrophils for stability of CD177 phenotype over time.
(PDF)

S3 Fig A-C. Sequencing read depth for exons 4, 5 and 7 (A-C). D-F. Genotyping results for CD177 determined by Amplifluor assays. G. Prevalence of genotypes in both cohorts. H. Prevalence of genotypes by ethnicity.
(PDF)

S4 Fig Nucleotide sequence alignment of CD177 exon 4 (A), 5 (B) and 7 (C) in human and mammal subsets. (PDF)

S5 Fig Linkage disequilibrium plots (expressed as r^2^ or D’) of *CD177-CD177P1* locus using East Asian (CHS) and Central European (CEU) population data from Genome1000 project. Figures were generated from Ensembl. 165 and 190 SNPs were examined in the two studies respectively. SNPs near exon 7 used in both studies were listed along the LD plots.
(PDF)

S6 Fig Phylogenetic summary of CD177 and CD177 related sequences from selected primates. (PDF)

S7 Fig Exonic structure of CD177 in humans, chimpanzee, macaque, wolf, mouse and rat. (PDF)

S8 Fig Amino acid sequence alignment to human CD177 with mouse CD177 and human orthologs. The PSI-Blast multiple sequence alignment was generated by hiden Markov model (HMM-HMM) matching with Phyre2 tools (www.sbg.bio.ic.au.uk/phyre2), colored by the properties of residues: Aromatic (dark green), Aliphatic (light green), charge (dark blue), hydroxylic (light blue), acidic (purple), basic (red) and sulfur containing (yellow) [52]. Genomic location of *CD177* and orthologs are indicated in the schematic structure of chromosome 19q13.2 –q13.31.
(PDF)

S9 Fig Amino acid sequence alignments of CD177 in humans, chimpanzee, macaque, wolf, mouse and rat. Dashes indicate absence of corresponding amino acid. Human exons are shown in alternating blue and black text. Lysine subject to substitution from gene conversion is shown in red.
(PDF)

S10 Fig Alignment of amino acids from first half (A) and second half (B) of mouse CD177 with human CD177. Identity and E values determined by BLAST.
(PDF)

S11 Fig Alignment of human *CD177* nucleotide sequence with *CD177P1*. Data was generated by Blastn *CD177* gDNA sequence from Ensembl with some small gaps manually aligned. The alignment suggests that *CD177P1* locus (43,372,742–43,379,123) should be expanded to 43,371,891–43,380,385, containing *CD177* homologs from intron 3 / 4 to the end of exon 9 including 3’UTR. Dark blue and black letters indicates alternative exons, light blue letters represent intron retention. Polymorphic nucleotides in exon 4, 5, and 7 identified from this work have been highlighted and revised.
(PDF)
