# Supplementary material for: Heterogeneity of Human Neutrophil CD177 Expression Results from CD177P1 Pseudogene Conversion
Source: PLoS Genet. 2016 May 26;12(5):e1006067. doi: 10.1371/journal.pgen.1006067 (PMC4882059; doi:10.1371/journal.pgen.1006067)
Supplement: S1 Table — (PDF) [file pgen.1006067.s001.pdf]

Supportive Information Table 1. Summary of previous reports of genetic variations associated with altered CD177 expression

	Major findings	Location (hg38)	n	Source material	Technique	Coverage of CD177	Limitation	Ref
1	Truncated transcripts cDNA655-773 & cDNA381-526	19:43359148-266 (intron5/6 retention); 19:43355515-566 (exon 4 alt 5') †	2 (CD177 deficient)	Neutrophil cDNA	RT-PCR, subcloning	CDS	No splice site mutation identified (gDNA not sequenced	30
2	cDNA 34C>G, 778A>C, and 1069G>A associated with lower %CD177+	19:43353706 (g.34); 19:43360262 (g.6590); 19:43361290 (g.7618) ‡	19 (CD177+)	Neutrophil cDNA	RT-PCR, Sanger sequencing	CDS	No CD177^{null} included	31
3	cDNA G42C associated to higher %CD177+	19:43353714 (g.43, 5'UTR) ‡	23 (CD177+)	WBC DNA	PCR, Sanger sequencing	Partial CD177 gDNA	Majority of exon 7 sequence missed	32
4	A793C & G1084A associated with lower CD177 expression	19:43360277 (g.6605); 19:43361305 (g.7633) ‡	135 healthy subjects	WBC cDNA	RT-PCR, Sanger sequencing	CDS	No CD177^{null} included	33
5	cDNA829A>T	19:43361169 (g.7497)	294 healthy Americans	cDNA & gDNA	Sanger sequencing	CDS and gDNA	Mechanism unknown	34
6	CD177 g.7497A>T (c787A>T)	19:43361169 (g.7497)	535 healthy subjects & 40 vasculitis patients	Neutrophil cDNA & gDNA	Deep sequencing	CDS and gDNA		Wu et al

†: genomic loci mapped from cDNA sequence;
‡: genomic loci mapped from cDNA numbers.

S1_Table
