# Supplementary material for: Heterogeneity of Human Neutrophil CD177 Expression Results from CD177P1 Pseudogene Conversion
Source: PLoS Genet. 2016 May 26;12(5):e1006067. doi: 10.1371/journal.pgen.1006067 (PMC4882059; doi:10.1371/journal.pgen.1006067)
Supplement: S2 Table — (PDF) [file pgen.1006067.s002.pdf]

Supportive Information Table 2. In silico analysis of *CD177* single nucleotide variations

Substitution	PolyPhen2 classification	PolyPhen2 Score	SIFT Classification	CADD Phred-like Score
A3P	Benign	0	Tolerated	0.029
H31L	Benign	0.007	Tolerated	0.001
L38L	Benign	0	Tolerated	0.466
P128A	Possibly damaging	0.717	Deleterious	2.455
V184G	Possibly damaging	0.909	Tolerated	8.61
D204N	Benign	0.004	Tolerated	0.178
M205R	Benign	0.007	Tolerated	1.265
M237T	Benign	0	Tolerated	0.256
L251I	Benign	0	Tolerated	3.396
G261A	Benign	0.061	Tolerated	7.193
T262T	Benign	0	Tolerated	0.533
K263X	---	---	Stop-gain	---
G264S	Possibly damaging	0.71	Tolerated	5.158
G264V	Probably damaging	0.999	Deleterious	6.646
T267A	Benign	0	Tolerated	0.755
A348T	Benign	0.029	Tolerated	2.021
G431R	Probably damaging	0.999	Deleterious	1.269
