# Supplementary figures and images for: Heterogeneity of Human Neutrophil CD177 Expression Results from CD177P1 Pseudogene Conversion
Source: PLoS Genet. 2016 May 26;12(5):e1006067. doi: 10.1371/journal.pgen.1006067 (PMC4882059; doi:10.1371/journal.pgen.1006067)

## CD177 Transcripts

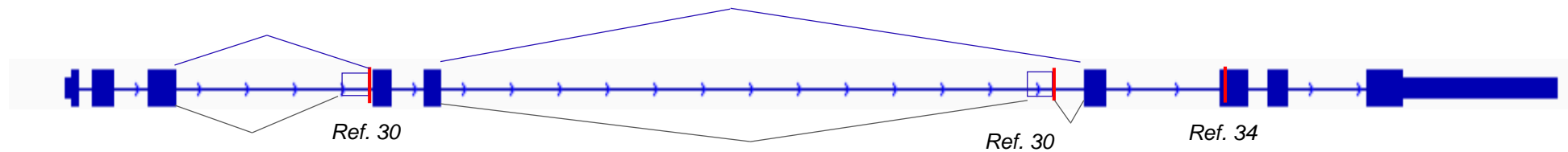

Supplement: S1 Fig — (PDF) [file pgen.1006067.s003.pdf]

CD177 distance tree view of primates

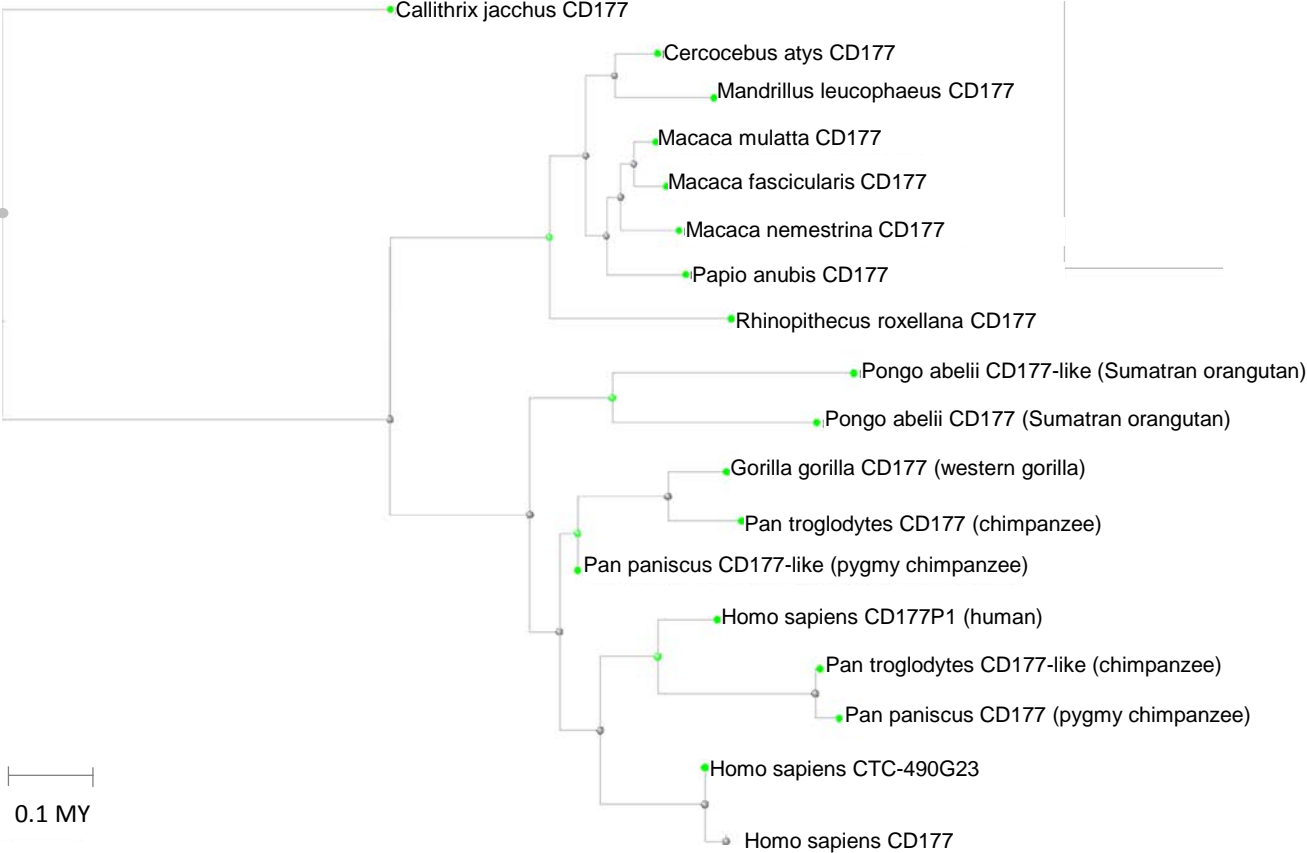

Supplement: S6 Fig — (PDF) [file pgen.1006067.s008.pdf]

## CD177 exon structure

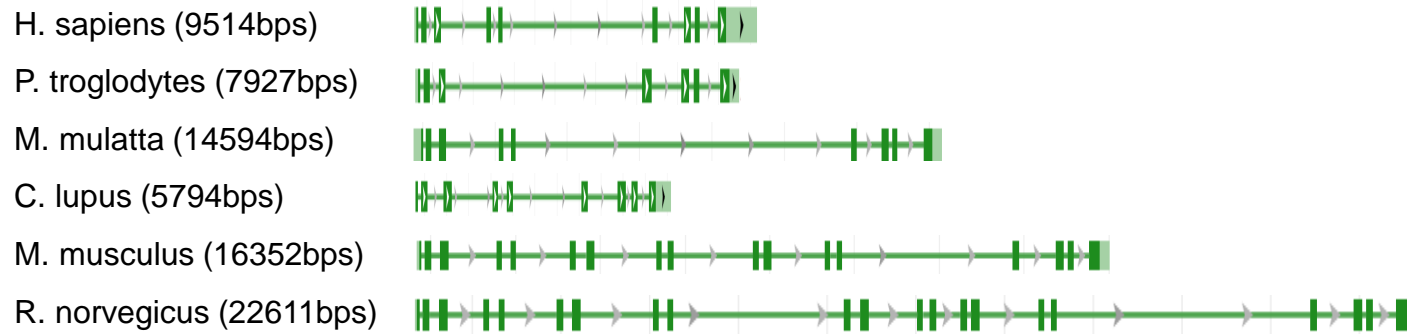

Supplement: S7 Fig — (PDF) [file pgen.1006067.s009.pdf]

Alignment to human CD177 protein

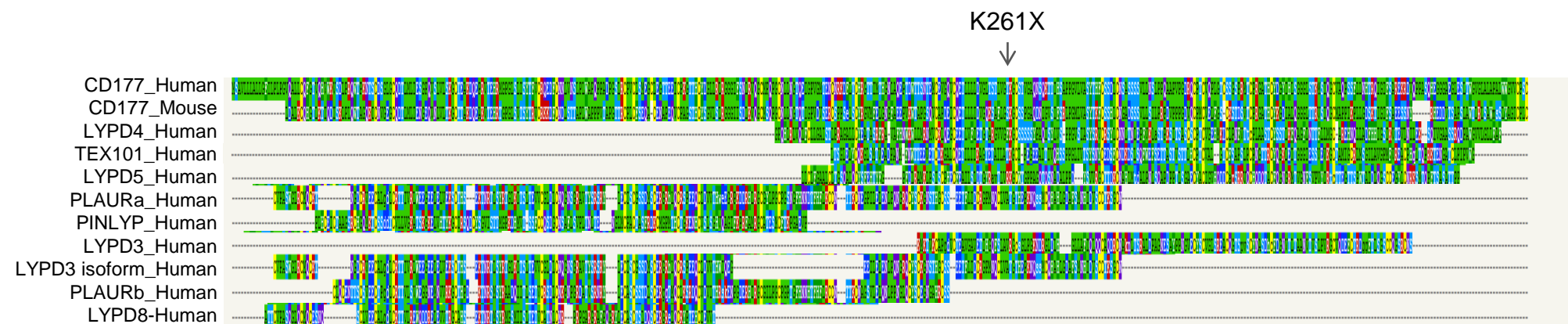

Gene location of human *CD177* and orthologs

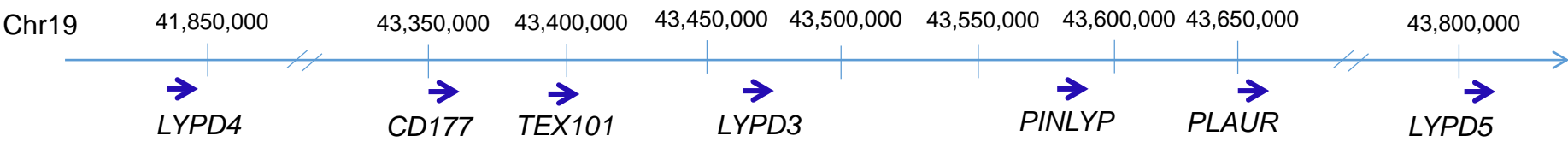

Supplement: S8 Fig — The PSI-Blast multiple sequence alignment was generated by hiden Markov model (HMM-HMM) matching with Phyre2 tools (www.sbg.bio.ic.au.uk/phyre2), colored by the properties of residues: Aromatic (dark green), Aliphatic (light green), charge (dark blue), hydroxylic (light blue), acidic (purple), basic (red) and sulfur containing (yellow) [52]. Genomic location of CD177 and orthologs are indicated in the schematic structure of chromosome 19q13.2 –q13.31. (PDF) [file pgen.1006067.s010.pdf]
