# Supplementary material for: Heterogeneity of Human Neutrophil CD177 Expression Results from CD177P1 Pseudogene Conversion
Source: PLoS Genet. 2016 May 26;12(5):e1006067. doi: 10.1371/journal.pgen.1006067 (PMC4882059; doi:10.1371/journal.pgen.1006067)
Supplement: S2 Fig — A. Ethnicity of the study subjects in the two cohorts. B. Prevalence of CD177 phenotypes in both cohorts. C-E. Analysis of neutrophils for stability of CD177 phenotype over time. (PDF) [file pgen.1006067.s004.pdf]

A **Ethical groups of study subjects**

Ethnicity	Cohort 2 (%)	Cohort 1 (%)
European (n=405)	70	80
Asian (n=57)	10	10
Australian (n=108)	19	10
African (n=5)	1	0

B **Prevalence of CD177 phenotypes (%)**

	CD177 phenotype			
	Null	Biomodal	High	Atypical
Cohort 2 (n=535)	2.6	24.7	65.4	7.3
Cohort 1 (n=40)	5.0	22.5	62.5	10

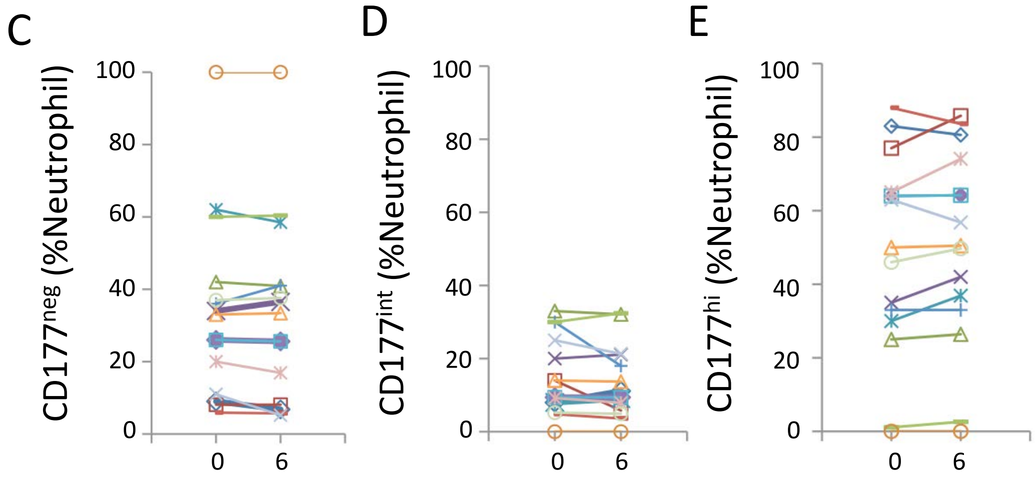

S2_Figure
