# Supplementary material for: Heterogeneity of Human Neutrophil CD177 Expression Results from CD177P1 Pseudogene Conversion
Source: PLoS Genet. 2016 May 26;12(5):e1006067. doi: 10.1371/journal.pgen.1006067 (PMC4882059; doi:10.1371/journal.pgen.1006067)
Supplement: S3 Fig — A-C. Sequencing read depth for exons 4, 5 and 7 (A-C). D-F. Genotyping results for CD177 determined by Amplifluor assays. G. Prevalence of genotypes in both cohorts. H. Prevalence of genotypes by ethnicity. (PDF) [file pgen.1006067.s005.pdf]

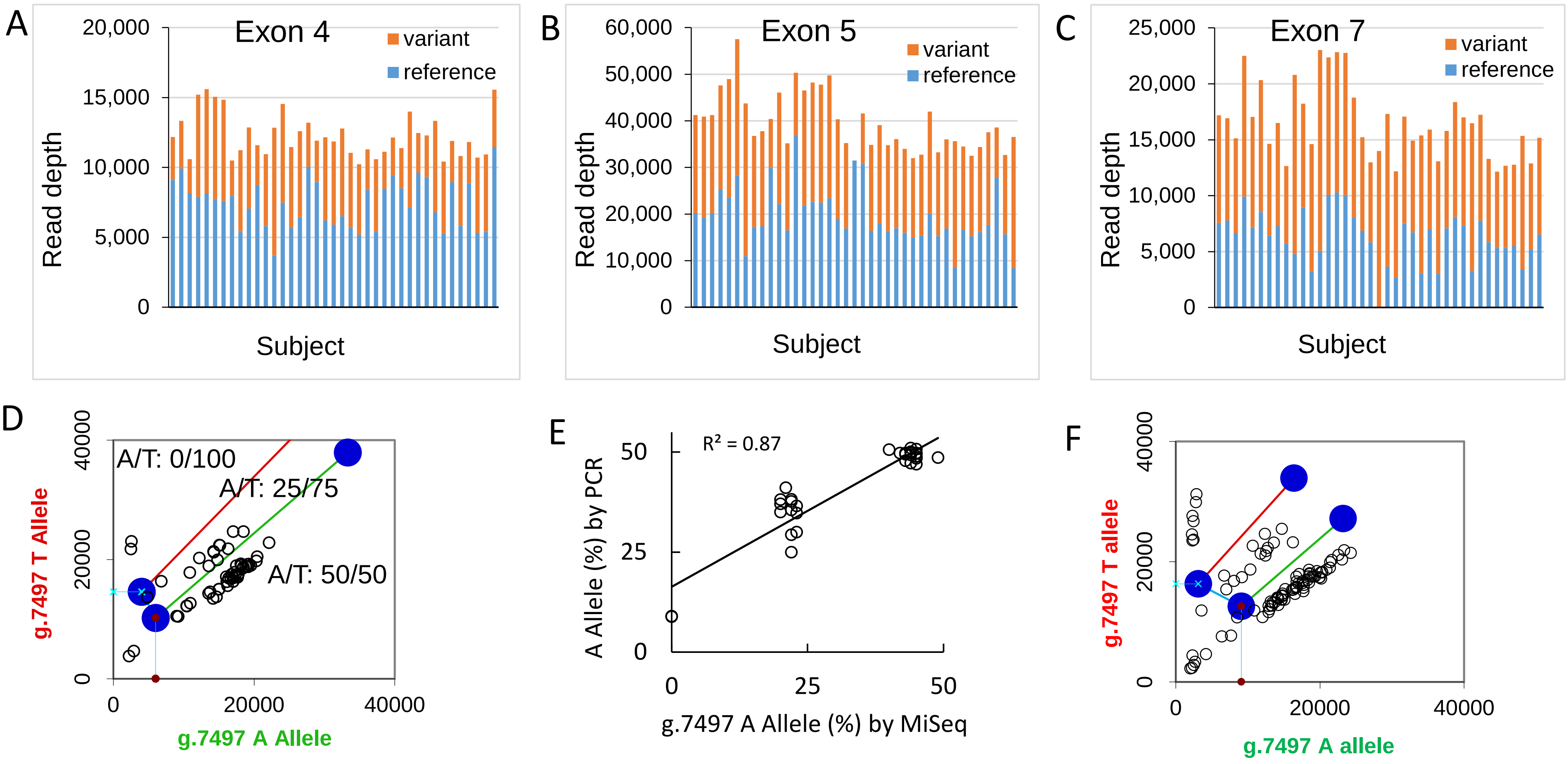

G Prevalence of CD177g.7497 genotypes (%)

	CD177 g.7497 A/T allele ratio		
	0/4	1/3	2/2
Cohort 2 (n=535)	2.6	19.5	77.9
Cohort 1 (n=40)	5.0	22.5	72.5

H Prevalence of CD177g.7497 genotypes by ethnicity

	g.7497 (A/T: 0/4) (%)	g.7497 (A/T: 1/3) (%)	g.7497 (A/T: 2/2) (%)
European (n=405)	2	19	79
Asian (n=57)	2	23	75
Australian (n=108)	5	20	75
African (n=5)	0	20	80

S3_Figure
