# Supplementary material for: Heterogeneity of Human Neutrophil CD177 Expression Results from CD177P1 Pseudogene Conversion
Source: PLoS Genet. 2016 May 26;12(5):e1006067. doi: 10.1371/journal.pgen.1006067 (PMC4882059; doi:10.1371/journal.pgen.1006067)
Supplement: S4 Fig — (PDF) [file pgen.1006067.s006.pdf]

A. Alignment CD177 Exon 4, 43,355,660 – 43,355,726 (67 bps)

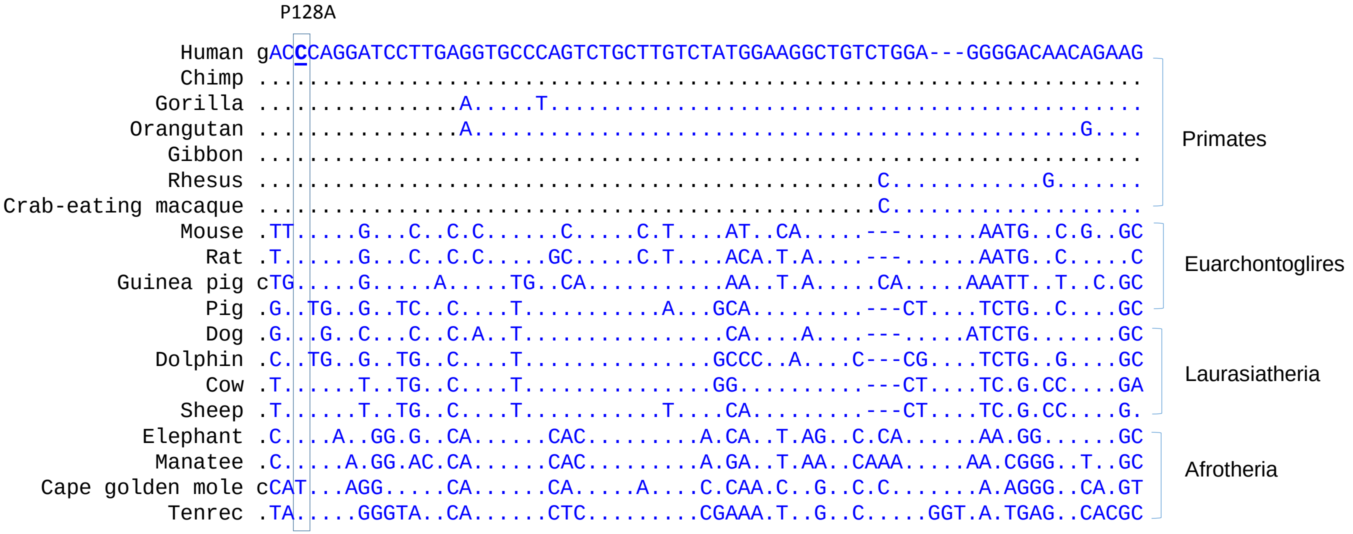

B. Alignment CD177 Exon 5, 43,356,033 – 43,356,109 (77 bps)

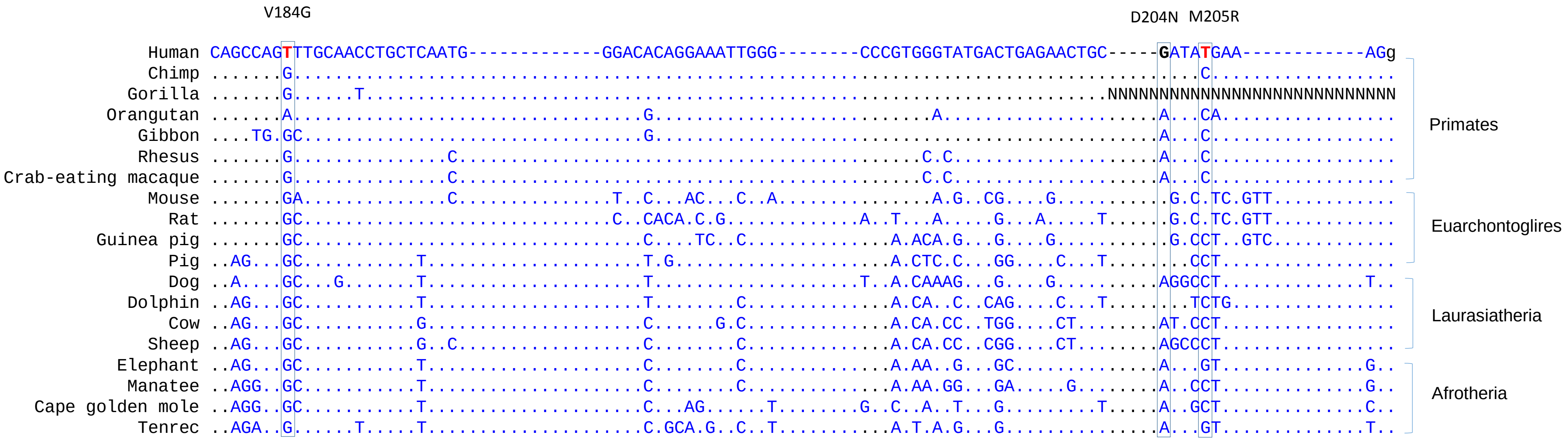

S4_Figure

C. Alignment CD177 exon 7,
43,361,142 – 43,361,195 (54 bps)

G261A T262T K263X G264S G264V T267A

```
            Human gGACTCACATC-AACCCTGGTGGGGACAAAAGGCT---G-----CAGCA---C------TGTTGGGGCTCAA
            Chimp ................................................G.......................
          Gorilla ....................A..C...C...A................G.......................
        Orangutan ...........................C...A................G.......................
           Gibbon ...........................C....................G.......................
           Rhesus ....G.......G.........T...GC....................G.......................
            Mouse ....AA.A.....G..T.CC......GT........................G.......CC......G..G
              Rat ....AA.A.....G....CC......GC........................G.......C....A..A..G
       Guinea pig ....AGCA.....CT...T.....A.GC........................G.......CC..A.AGA..G
              Pig ....A..G.....CT...CC...CA.GC........................A......G.CCA..A.A..G
              Dog ....A..A......T.A.A..A..A.GC.....T..................A......G.GCA..A.A..G
          Dolphin ....C..G....GCT...........GC........................A......G.CCA..A.A.CG
              Cow ....C..GG...G.T....C......GC....................C...A......G..CAATA-----
            Sheep ....C..GG...G.T....C....A.GC....................C...A......G..CA.AA-----
         Elephant ....A..A..T..CT...AC......GC................TGC.T...........CC....A.AA.G
          Manatee ....A..A.C...CT...........AC................TGC.T..........CTC...AA.A..G
Cape golden mole ..TA..A....CCTT..AC......GC.....T..........T.C.T...T......GCC....A.A.. .
           Tenrec ...AAT...G...GT...AT......GC..................T.T...TGCCGGGGCCG...C.A..G
```

Primates

Euarchontoglires

Laurasiatheria

Afrotheria

S4_Figure (continue)
