# Supplementary material for: Heterogeneity of Human Neutrophil CD177 Expression Results from CD177P1 Pseudogene Conversion
Source: PLoS Genet. 2016 May 26;12(5):e1006067. doi: 10.1371/journal.pgen.1006067 (PMC4882059; doi:10.1371/journal.pgen.1006067)
Supplement: S5 Fig — Figures were generated from Ensembl. 165 and 190 SNPs were examined in the two studies respectively. SNPs near exon 7 used in both studies were listed along the LD plots. (PDF) [file pgen.1006067.s007.pdf]

CD177/CD177P1
linkage disequilibrium

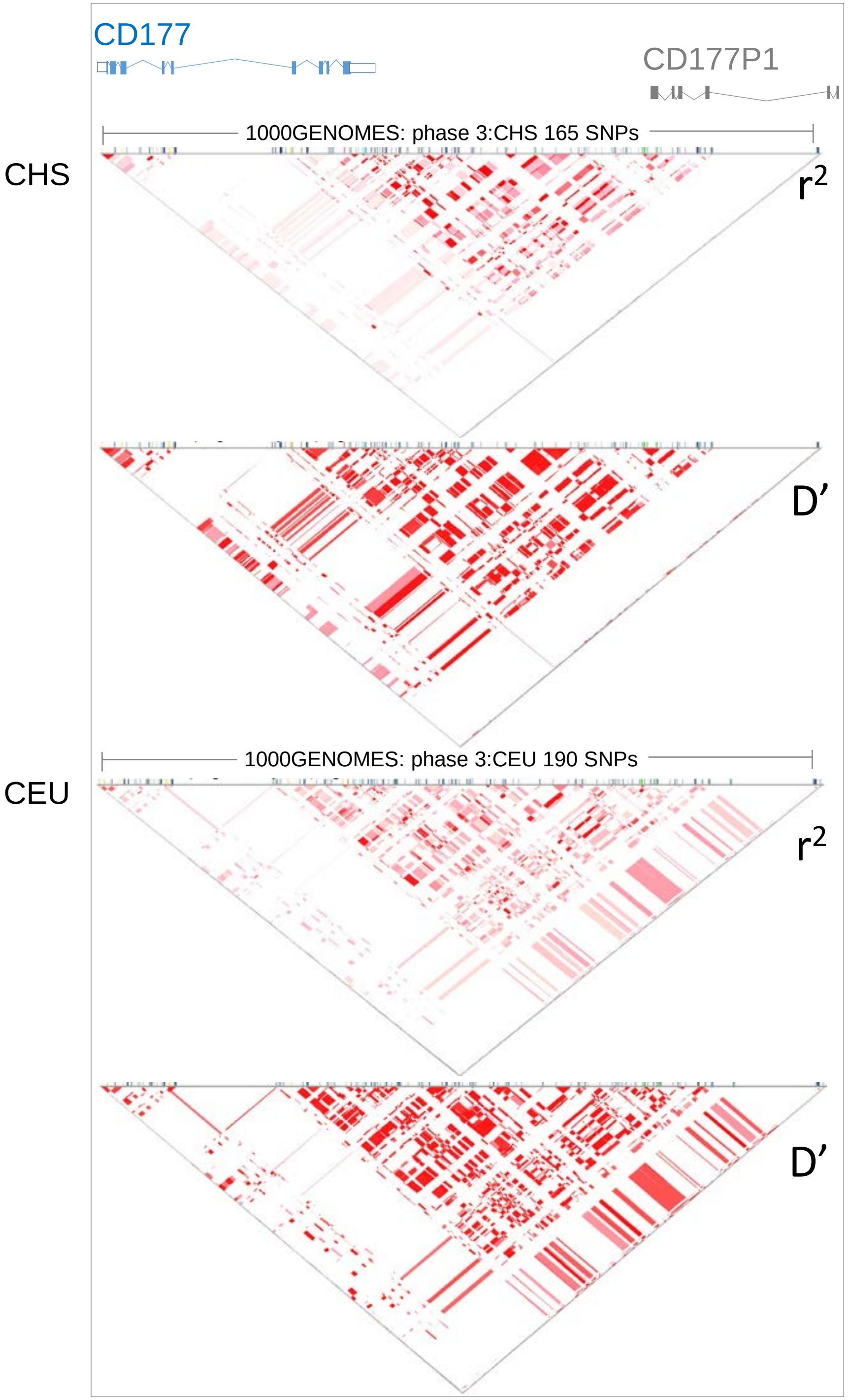

SNPs near exon 7 of *CD177* and *CD177P1*

SNP	Location	Coordinate
rs373560341	nc-transcript	43360787
rs62114799	intron 6/7	43360937
rs12977607	intron 6/7	43360940
rs12977173	intron 6/7	43360966
rs12977179	intron 6/7	43360977
rs371812801	intron 7/8	43361392
rs113094109	intron 8/9	43361650
rs28464741	intron 8/9	43361688
rs3745996	nc-transcrpt (P1 ex8)	43373242
rs3745997	nc-transcrpt (P1 ex8)	43373254
rs201309678	nc-transcrpt (P1 ex8)	43373315
rs201963773	nc-transcrpt (P1 ex8)	43373332
rs201754481	P1 intron 7/8	43373373
rs3745998	P1 intron 7/8	43373388
rs536047768	P1 intron 6/7	43373803
rs35996688	P1 intron 6/7	43373988

S5_Figure
