# Supplementary material for: Heterogeneity of Human Neutrophil CD177 Expression Results from CD177P1 Pseudogene Conversion
Source: PLoS Genet. 2016 May 26;12(5):e1006067. doi: 10.1371/journal.pgen.1006067 (PMC4882059; doi:10.1371/journal.pgen.1006067)
Supplement: S9 Fig — Dashes indicate absence of corresponding amino acid. Human exons are shown in alternating blue and black text. Lysine subject to substitution from gene conversion is shown in red. (PDF) [file pgen.1006067.s011.pdf]

Alignment of human CD177 protein

```
H.sapiens        1 MSAVLLLALLGFILPLPGVQALLCQFGTVQHVWKVSDLPRQ-------------------------------------------------------------------------------------------------------------  41
P.troglodytes    1 MSPVLLLALLGVTLPLPGVQALFCQLGTNHRVWKVSDLPRQ-------------------------------------------------------------------------------------------------------------  41
M.mulatta        1 ----------MHCVP-PGEFPAGCP-GADAR----PKLPLH-------------------------------------------------------------------------------------------------------------  25
C.lupus          1 MSPALQLAFLGMTLMLSRVQALTCQSGTHNSLRDVLELPLE-------------------------------------------------------------------------------------------------------------  41
M.musculus       1 MNSIPVLTLLGVTALLPCVPALTCQKSSAQAVRNVAELPLWWGAGEKTCEVSEGCQDLIMLLYNGPKVNLVIIKGCTEVEDQEPKVIWLRTGPGLSVVSYTRVCRHGDLCNDVNSTKILEELPTPTVPGSLRCPLCLSNDSCENAPEQV  150
R.norvegicus     1 MNPMPVLTLLGVSTLLPCVPALICQRGRIQTVRNESELPLE-WGTGEKACEVGEGCQDVVMLLHNGPLINLVIIKGCIKAEDQEPRVTWLSTGPGLSVVSYTHVCRHGDLCNNASSTRILEDLSTPTVPGSLRCPLCLSNNDCENAPEQV 149

H. sapiens         ------------------------------------------------------------------------------------------------------------------------------------------------------
P.troglodytes      ------------------------------------------------------------------------------------------------------------------------------------------------------
M.mulatta          ------------------------------------------------------------------------------------------------------------------------------------------------------
C.lupus            ------------------------------------------------------------------------------------------------------------------------------------------------------
M.musculus     151 CPVGSTHCYDGVLRLRGDGIRTNLKVQGCMAQPDCNLLNGTQAIGTLYMSENCDL-IGPQALDCNSGSLETVRNVSDLHLSWTTGWQTCEAGEGCYETVMLIQNGHEFHMVLTKGCTRDMNKKARLTRHRTGPGISIVSYVHVCRDRDFC 299
R.norvegicus   150 CPVGSIYCYNGVLRLRGEGIRTNLKVQGCMAQPGCNLLNGTTAVGTLDMSENCGLQLGPQALDCNSASLDIVKDVSDLHLSWTTGWKTCEADEGCYETVMLIQNGQEIHMALTKGCTSSVNREAQLTRHRTGPGISIVSYVHVCRNGDFC 299

H. sapiens         --------------------------------------------------------------------------------------------------------------------------WTPKNTSCDSGLGCQDTLMLIESGPQVS  69
P.troglodytes      --------------------------------------------------------------------------------------------------------------------------WTPKNTSCDSGLGCQDTLMLIESG----  65
M.mulatta          --------------------------------------------------------------------------------------------------------------------------WTPKEISCDSGLGCQDTLVLIESGPQVS  53
C.lupus            --------------------------------------------------------------------------------------------------------------------------WTAGQESCEDGWGCQDTLILIENGPQVN  69
M.musculus     300 NDLSTTDPLWTPPPDTELGTLRCRHCLSTGSCVSASELVCPAGSTHCYSGVLSLRGGGVISDLKVQGCI--SQSQPGCNLLNGTQTIGPVDVREDCGLQLDALKCQHGTLKTIQDISKLPLQWTAGQKICNVGEGCQDTLMLIENGEQVN 447
R.norvegicus   300 NDLSSTETLWIPPPDTVPGTLRCPHCLSTRDCENAPEQVCPEG-THCYNGVLRLRGGGFISDLKVQGCTSQSQSQSDCNLLNGIQTIGPIKLHESCGFHLESLKCQHGTLEAVQHISKLPLQWTAEQKTCNVGEGCQDTLVIIENGEHVN 448

H.sapiens       70 LVLSKGCTEAKDQEPRVTEHRMGPGLSLISYTFVCRQEDFCNNLVNSLPLWAPQPPADPGSLRCPVCLSMEGCLEGTTEEICPKGTTHCYDGLLRLRGGGIFSNLRVQGCMPQPVCNLLNGTQEIGPVGMTENC-------DMKDFLTCH 212
P.troglodytes   66 ---EKGCTEAKDQEPRVTEHRMGPGLSLISYTFVCRPEDFCNNLVNSLPLWAPQPPQ-------VACV-----------------------------GGSWGLSLRVPASWDR--SPLL--------------C-------D-GHFLTCH 152
M.mulatta       54 LVLSKGCTEAKDREPRVTEHRMGPGLSVLSYTYVCRHKDFCNNLVTTAPLWTPQPPADPGSLRCPVCLSMEGCPEGTTEEMCPKGTTHCYNGLLRLRGGGIFSNLRVQGCLPQPGCNLLNGTQEIGPVRMTENC-------NTKDFLTCH 196
C.lupus         70 VVISKGCTAAADQDVLIREHRSGPGLSILSYTHVCREKDNCNSLSTTLPLWSLPSTTGPGSLRCPVCLSTEDC-ESATELACPAGSTHCYRGAVWIRGGNIFQILRVQGCMSQPGCDLLNGTQEIGSIKVSEDCRPNGECQGHKNFSDLS 218
M.musculus     448 LVLTKGCTTAKDQEAKVTEHRTGPGLSVTSYTRVCRKKDFCNDLSTTAPLWAPPPVTAPGTTRCPLCFSEQAC-ENAPEQVCPAGSTHCYSGVLSLRGGGIISDLKVQGCMSQPGCNLLNGTQTIGPVDVSERCSPP----SETTELSCY 592
R.norvegicus   449 LVLTKGCTAAEDHKAKVTEHRTGPGLAVISYTRVCREKDLCNDLSTTVPLWAPPPVTAPGNTRCPLCFTAQAC-ENAPEQFCPAGSTHCYSGVLSLRGGEIISKLKVQGCMSQPGCNLLNGTQKIGPVDVSEDCSPR----SNS--LTCH 591

H.sapiens      213 RGTTIMTHGNLAQEPTDWTTSNTEMCEVGQVCQETLLLLDVGLTSTLVGTKGCSTVGAQNSQKTTIHSAPPGVLVASYTHFCSSDLCNSASSSSVLLNSLPPQAAPVPGDRQCPTCVQPLGTCSSGSPRMTCPRGATHCYDGYIHLSGGG 362
P.troglodytes  153 RGITFMTHTHNLAQEPTDWTTSNTEMCEAGQVCQETLLLVDVGLTSTLVGTKGCSAVGAQNSQKTTIHSAPPGVLVASYTHFCSSDLCNSASSSSVLLNSLPPQAAPVPGDRQCPTCVQPLGTCSSGSPQMTCPRGATHCYDGYIHLSGGG 302
M.mulatta      197 RGNSLQKGDNLAQVPIGWTVSNTVMCEAGQVCQETLLLIDVGRTSTLVWSKGCSAVGAQNSQKTSIHSAPPGVLVASYAHFCSSDLCNGASSSSVLLNSLPPQGAPEPGDQQCLTCVQPLATCSSDSPVKTCPRGTTHCYDGYISLSGGG 346
C.Lupus        219 RGIMLHSHQHLSQTPIKWTPNNYELCNIGEVCQETLLLIDVGHKSIIVGSKGCSKGRTQDAPTITIHSAPPGVLVASYAHFCSSNRCNRASSTSVLLNSLPHSAAPAPGDLQCPVCLQIFGSCPENPEKVTCPSGTTHCYSGYITLREGG 368
M.musculus     593 RGVMFELGNGFAEEPVKWTAPGSQVCAPDEICQETLLLIDVGQKSAFLGSKGCSSPGAQDNIGVSIFSRLPGMLVASYTKFCSSHLCNGADSSSVLLSILPRPDVPPPGDVQCPMCVELFGSC-KSTDSVTCPRGATHCYKGDIALQGGG 741
R.norvegicus   592 RGTMLKIGNGFAEKAVEWTPLSSQVCEPDEICQETLLLIDVGQKSALLGSKGCSSLGAQNDVGVSIFSRPPGMVVASYTRFCSSNLCNAASSTSVLLSNLPRPEVLPPGSVKCPVCVQLFRSCFQTSDFVTCPRGATHCYRGDIALRGGG 741

H.sapiens      363 LSTKMSIQGCVAQPSSFLLNHTRQIGIFSAREKRDVQPPASQHEGGG--AEGLESLTWGVGLALAPALWWGVVCPSC 437
P.troglodytes  303 LTSTMSIQGCVAQPSSFLLNHTRQIGIFSAREKRGVQPPASQPEGGG--AEGLESLTWGVGLALAPALWWGAVCPSC 377
M.mulatta      347 VTTTMGIQGCVAQPSSSLLNHTRQIGIFSVREKGEEEPPAPHSERGG--AGGLESLTWGVGLALALALWWGALCPSC 421
C.Lupus        369 IYSALDIQGCMTQGSSSLLNHARTIGVFSVME--DSKKSRTQFSR--------------LGRSLHK----------- 418
M.musculus     742 LTTRVSIQGCMAPPIKPLLGDSKTIGIFSAEESSNYRHEDDVTSAPSL-AWTLRLSAWMLGLSALLSSLYAGICPLC 817
R.norvegicus   742 LSSTLSIQGCMASPVKSLLGNSKTIGILSAKESSENERGDEDDEKPLLDGASASSPASMLGLLVLLSSLCAGICPLH 818
```

S9_Figure
