# Supplementary material for: Heterogeneity of Human Neutrophil CD177 Expression Results from CD177P1 Pseudogene Conversion
Source: PLoS Genet. 2016 May 26;12(5):e1006067. doi: 10.1371/journal.pgen.1006067 (PMC4882059; doi:10.1371/journal.pgen.1006067)
Supplement: S10 Fig — Identity and E values determined by BLAST. (PDF) [file pgen.1006067.s012.pdf]

A **Human CD177 aligned to first half of mouse protein (43.03% identity, E value: 2e-73):**

```
Human        17 PGVQALLCQFGTVQHVWKVSDLP-RQWTPKNTSCDSGLGCQDTLMLIESGPQVSLVLSKG    75
Mouse-a      17 PCVPALTCQKSSAQAVRNVAELPLRWWGAGEKTCEVSEGCQDLIMLLYNGPKVNLVIIKG    76

Human        76 CTEAKDQEPRVTEHRMGPGLSLISYTFVCRQEDFCNNLVNSLPLWAPQP-PADPGSLRCP   134
Mouse-a      77 CTEVEDQEPKVIWLRTGPGLSVVSYTRVCRHGDLCND-VNSTKILEELPTPTVPGSLRCP   135

Human       135 VCLSMEGCLEGTTEEICPKGTTHCYDGLLRLRGGGIFSNLRVQGCMPQPVCNLLNGTQEI   194
Mouse-a     136 LCLSNDSC-ENAPEQVCPVGSTHCYDGVLRLRGDGIRTNLKVQGCMAQPDCNLLNGTQAI   194

Human       195 GPVGMTENCDM--KDFLTCHRGTTIMTHGNLAQEPTDWTTSNTEMCEVGQVCQETLLLLD   252
Mouse-a     195 GTLYMSENCDLIGPQALDCNSG-SLETVRNVSDLHLSWTT-GWQTCEAGEGCYETVMLIQ   252

Human       253 VGLTSTLVGTKGCSTVGAQNSQKTTIHSAPPGVLVASYTHFCSSDLCNSASSSVLLNSL   312
Mouse-a     253 NGHEFHMVLTKGC-TRDMNKKARLTRHRTGPGISIVSYVHVCRDRDFCNDLSTTDPLWTP   311

Human       313 PPQAAPVPGDRQCPTCVQPLGTCSSGSPRMTCPRGATHCYDGYIHLSGGGLSTKMSIQGC   372
Mouse-a     312 PPDTE--LGTLRCRHCLS-TGSCVSAS-ELVCPAGSTHCYSGVLSLRGGGVISDLKVQGC   367

Human       373 V--AQPSSFLLNHTRQIGIFSAREKRDVQPPASQHEGGGAEGLESLT-----WGVG      421
Mouse-a     368 ISQSQPGCNLLNGTQTIGPVDVREDCGLQLDALKCQHGTLKTIQDISKLPLQWTAG      423
```

B **Human CD177 aligned to second half of mouse protein (54.17% identity, E value: 1e-101):**

```
Human        19 VQALLCQFGTVQHVWKVSDLPRQWTPKNTSCDSGLGCQDTLMLIESGPQVSLVLSKGCTE    78
Mouse-b     397 LDALKCQHGTLKTIQDISKLPLQWTAGQKICNVGEGCQDTLMLIENGEQVNLVLTKGCTT   456

Human        79 AKDQEPRVTEHRMGPGLSLISYTFVCRQEDFCNNLVNSLPLWAPQPPADPGSLRCPVCLS   138
Mouse-b     457 AKDQEAKVTEHRTGPGLSVTSYTRVCRKKDFCNDLSTTAPLWAPPPVTAPGTTRCPLCFS   516

Human       139 MEGCLEGTTEEICPKGTTHCYDGLLRLRGGGIFSNLRVQGCMPQPVCNLLNGTQEIGPVG   198
Mouse-b     517 EQAC-ENAPEQVCPAGSTHCYSGVLSLRGGGIISDLKVQGCMSQPGCNLLNGTQTIGPVD   575

Human       199 MTENC---DMKDFLTCHRGTTIMTHGNLAQEPTDWTTSNTEMCEVGQVCQETLLLLDVGL   255
Mouse-b     576 VSERCSPPSETTELSCYRGVMFELGNGFAEEPVKWTAPGSQVCAPDEICQETLLLIDVGQ   635

Human       256 TSTLVGTKGCSTVGAQNSQKTTIHSAPPGVLVASYTHFCSSDLCNSASSSVLLNSLPPQ   315
Mouse-b     636 KSAFLGSKGCSSPGAQDNIGVSIFSRLPGMLVASYTKFCSSHLCNGADSSSVLLSILPRP   695

Human       316 AAPVPGDRQCPTCVQPLGTCSSGSPRMTCPRGATHCYDGYIHLSGGGLSTKMSIQGCVAQ   375
Mouse-b     696 DVPPPGDVQCPMCVELFGSCKS-TDSVTCPRGATHCYKGDIALQGGGLTTRVSIQGCMAP   754

Human       376 PSSFLLNHTRQIGIFSAREKRDVQPPASQHEGGGAEGLESLTWGVGLA              423
Mouse-b     755 PIKPLLGDSKTIGIFSAEESSNY-----RHE-DDVTSAPSLAWTLRLS              796
```

S10_Figure
