# Supplementary material for: Heterogeneity of Human Neutrophil CD177 Expression Results from CD177P1 Pseudogene Conversion
Source: PLoS Genet. 2016 May 26;12(5):e1006067. doi: 10.1371/journal.pgen.1006067 (PMC4882059; doi:10.1371/journal.pgen.1006067)
Supplement: S11 Fig — Data was generated by Blastn CD177 gDNA sequence from Ensembl with some small gaps manually aligned. The alignment suggests that CD177P1 locus (43,372,742–43,379,123) should be expanded to 43,371,891–43,380,385, containing CD177 homologs from intron 3 / 4 to the end of exon 9 including 3’UTR. Dark blue and black letters indicates alternative exons, light blue letters represent intron retention. Polymorphic nucleotides in exon 4, 5, and 7 identified from this work have been highlighted and revised. (PDF) [file pgen.1006067.s013.pdf]

## S11_Figure: CD177/CD177P1 Sequence Alignment

```
CD177    ENSG00000204936:           1 AAAGGACTTGTTTCCTGCTGAAAAAGCAGAAAGAGATTACCAGCCACAGACGGGTCATGA ENSG00000204936:     60

CD177    ENSG00000204936:          61 GCGCGGTATTACTGCTGGCCCTCCTGGGGTTCATCCTCCCACTGCCAGGTGAGTGATGAG ENSG00000204936:    120

CD177    ENSG00000204936:         121 CCCAGCCTGGAGGAGATTCCCTGGAGGCCGGGCAAGGGAACCCTGCTGAGATGGATTTGC ENSG00000204936:    180

CD177    ENSG00000204936:         181 TCTTGCCACTCCAGGAGTGCAGGCGCTGCTCTGCCAGTTTGGGACAGTTCAGCATGTGTG ENSG00000204936:    240

CD177    ENSG00000204936:         241 GAAGGTGTCCGACCTGCCCCGGCAATGGACCCCTAAGAACACCAGCTGCGACAGCGGCTT ENSG00000204936:    300

CD177    ENSG00000204936:         301 GGGGTGCCAGGACACGTTGATGCTCATTGAGAGCGGTGAGAAGGCCCTGGCGTGCAGAGA ENSG00000204936:    360

CD177    ENSG00000204936:         361 CCCCGCCCTGTCCCCTCAGTCCCTTGATCCCTGTGCAGGGACCCGGGAGCCACCCCTCCG ENSG00000204936:    420

CD177    ENSG00000204936:         421 GGGGATCGACTCCTAGGGTCCCGGTGATCCCCTTTCCAGCCTCTCAGCCTACGCCGTGTA ENSG00000204936:    480

CD177    ENSG00000204936:         481 GCAGCGTCTCCCTCCAGGACCCTGGAGGCCTGACCTCCATCCTCGCTTGCCTCCCTCTTT ENSG00000204936:    540

CD177    ENSG00000204936:         541 CGGTCCAGGACCCCAAGTGAGCCTGGTGCTCTCCAAGGGCTGCACGGAGGCCAAGGACCA ENSG00000204936:    600

CD177    ENSG00000204936:         601 GGAGCCCCGCGTCACTGAGCACCGGATGGGCCCCGGCCTCTCCCTGATCTCCTACACCTT ENSG00000204936:    660

CD177    ENSG00000204936:         661 CGTGTGCCGCCAGGAGGACTTCTGCAACAACCTCGTTAACTCCCTCCCGCTTTGGGCCCC ENSG00000204936:    720

CD177    ENSG00000204936:         721 ACAGCCCCCAGCAGGTGCCTGCGGGAGGGTCGGGAGGAGAGGGAGGGGCTGCTAGAAGGG ENSG00000204936:    780

CD177    ENSG00000204936:         781 GATCCGCTGAGCACAGAGGGGCTGTTACGGAG                             ENSG00000204936:    812

(Alternative 3′ acceptor of exon 3)
CD177    ENSG00000204936:         813 TCCCTCCCACCCTCGCTCGCTATCCCGACCCTCGCTGGCTCCATCCCTCCCCTGACTGCT ENSG00000204936:    872
                                      ||||||||||||||||||||||||  | |||||||| |||||||||||||||||||||||
CD177P1                   19:43380285 TCCCTCCCACCCTCGCTCGCTATCGTGCCCCTCGCTCGCTCCATCCCTCCCCTGACTGCT              19:43380226

CD177    ENSG00000204936:         873 CCCTGACCATCGCCCCGCCCCGCTCCCTTTCCATCCCTCCCCACTCACTCCCGATCCCTC ENSG00000204936:    932
                                      ||||| |   ||||||||||| || |||||||||||||||||||||||||||||||||||
CD177P1                   19:43380225 CCCTGGCAGCCGCCCCGCCCCCCTTCCTTTCCATCCCTCCCCACTCACTCCCGATCCCTC              19:43380166

CD177    ENSG00000204936:         933 CCACCGACCT--                                                 ENSG00000204936:    942
                                      ||||||||||
CD177P1                   19:43380165 CCACCGACCTCG                                                               19:43380154

CD177    ENSG00000204936:         943 GCCACCCGGGAATCCCCGCACCCCTCCTCTTCTAAGAGCAAACAGGACGGATTTCTGATA ENSG00000204936:   1002
                                      ||||||||||||||||||||||||||||||||||||||||||||||||||||||||||||
CD177P1                   19:43380153 GCCACCCGGGAATCCCCGCACCCCTCCTCTTCTAAGAGCAAACAGGACGGATTTCTGATA              19:43380094

CD177    ENSG00000204936:        1003 TGAAATCACCATTAACTGGGTTATCCTGCATTCTTTTTTTATTCTGTTGCGTTTGGTTCA ENSG00000204936:   1062
                                      |||||| |||||||||||||||||||||||||| |||| |||||||||||||||||||||
CD177P1                   19:43380093 TGAAATTACCATTAACTGGGTTATCCTGCATTCCTTTTCTATTCTGTTGCGTTTGGTTCA              19:43380034

CD177    ENSG00000204936:        1063 AGAAGTTCTCCTGCAGGGTTTTGCTATTTCCTCTTCCTAAGGGAGACTGAACTGTAACT ENSG00000204936:   1122
                                      ||||||||||||||||||||||||| ||||||||||||||||||||||||||||||||||
CD177P1                   19:43380033 AGAAGTTCTCCTGCAGGGTTTTGCTGTTTCCTCTTCCTAAGGGAGACTGAACTGTAACT              19:43379974

CD177    ENSG00000204936:        1123 TTCCACTTTCCTTGTCTGGTGTTATAAAGAGTCTAGGGAGATATTTTGCGCTTCTTTTAT ENSG00000204936:   1182
                                      ||||||||||||||||||||||||||||||||||||||||||||||||||||||||||||
CD177P1                   19:43379973 TTCCACTTTCCTTGTCTGGTGTTATAAAGAGTCTAGGGAGATATTTTGCGCTTCTTTTAT              19:43379914

CD177    ENSG00000204936:        1183 TGGAACAGCTTGTGAACACAGAAGTCTCCTGTTCTTGGAGGTTTAGTAGCGCTTGCAGGT ENSG00000204936:   1242
                                      ||||||||||||||||||||||||||||||||||||||||| |||||||| |||||||||
CD177P1                   19:43379913 TGGAACAGCTTGTGAACACAGAAGTCTCCTGTTCTTGGAGGCTTAGTAGCACTTGCAGGT              19:43379854

CD177    ENSG00000204936:        1243 GAAGGCACTGGCTCGTTCCAGGGCACCCCCTCATTCCCTTGACCAATGAGTCTGCTTCTG ENSG00000204936:   1302
                                      ||||||||||||||||||||||||||||||||||||||||||||||||||||||||||||
CD177P1                   19:43379853 GAAGGCACTGGCTCGTTCCAGGGCACCCCCTCATTCCCTTGACCAATGAGTCTGCTTCTG              19:43379794

CD177    ENSG00000204936:        1303 TGTCTCCCCTCGGAAGGCCAGACTTTGTTGAAAGGTTCAGACTTGACCATGTCTGAGTCT ENSG00000204936:   1362
                                      ||||||||||||||||||||||||||||||||||||||||||||||||||||||||||||
CD177P1                   19:43379793 TGTCTCCCCTCGGAAGGCCAGACTTTGTTGAAAGGTTCAGACTTGACCATGTCTGAGTCT              19:43379734

CD177    ENSG00000204936:        1363 ACATTTGGCCTCAGCAGGCCCCTCCTCCACCCTGCCCACCCCCCACCCCCACTTGCCACC ENSG00000204936:   1422
                                      ||||||||||||||||||||||||||||||||||||||||||||||||||||||||| ||
CD177P1                   19:43379733 ACATTTGGCCTCAGCAGGCCCCTCCTCCACCCTGCCCACCCCCCACCCCCACTTGCCCCC              19:43379674

CD177    ENSG00000204936:        1423 CAAGAC                                                       ENSG00000204936:   1428
                                      ||||||
CD177P1                   19:43379673 CAAGAC                                                                     19:43379668

CD177    ENSG00000204936:        1429 CAAGACACCCCCCCTTTTCTTTTCTTTTTTTTT                            ENSG00000204936:   1461
                                             ||| |||
CD177P1                   19:43379667 -------CCCTCCC-------------------                                          19:43379661

CD177    ENSG00000204936:        1462 TTTTTTTTTTCTGAGAAAGCGTCTCGCTGTGTCGACCAGGCTGGAGTGCAGTGGCGCAAT ENSG00000204936:   1521
                                      |||||||||||||||||||||||||||||||| ||||||||| |||||||||||||||||
CD177P1                   19:43379660 TTTTTTTTTTCTGAGAAAGCGTCTCGCTGTGTTGACCAGGCTAGAGTGCAGTGGCGCAAT              19:43379601

CD177    ENSG00000204936:        1522 CTCGGCTCACTGCAACCTCCGACTCCCGGGTTCAAGCGATTCTCCTGCGTCAGCCTCCCA ENSG00000204936:   1581
                                      ||||||||||||||||||||||||||| ||||||||||||||||||| |||||| |||||
CD177P1                   19:43379600 CTCGGCTCACTGCAACCTCCGACTCCCAGGTTCAAGCGATTCTCCTGCCTCAGCGTCCCA              19:43379541

CD177    ENSG00000204936:        1582 CGTAGCAGGGACTAGAGGAGCCTGCCACCACGCCCGGCTAATTTTTGTATTTTTAGTAGA ENSG00000204936:   1641
                                       ||||| || ||| ||||||||  ||||||||||||||||||||||||||||||||||||
CD177P1                   19:43379540 AGTAGCTGGAACTGGAGGAGCCCACCACCACGCCCGGCTAATTTTTGTATTTTTAGTAGA              19:43379481

CD177    ENSG00000204936:        1642 GACAGGGTTTCACTATGTTGCCCAGGCTGGTCTTGAACTCCTGACCTCGTGATCCACCTG ENSG00000204936:   1701
                                      ||||| |||||||||||||| |||||||||||||||| |||||||||||||||||| |||
CD177P1                   19:43379480 GACAGAGTTTCACTATGTTGGCCAGGCTGGTCTTGAATTCCTGACCTCGTGATCCATCTG              19:43379421

CD177    ENSG00000204936:        1702 CCTCAGCCTCCCAAAGTGCTGGGATTACAGGTGTGAGTCACCACACCCAGCCAAGACCAC ENSG00000204936:   1761
                                      ||||||||||||||||||||||||||||||||||||| ||||||||||||||||||||||
CD177P1                   19:43379420 CCTCAGCCTCCCAAAGTGCTGGGATTACAGGTGTGAGCCACCACACCCAGCCAAGACCAC              19:43379361
```

```
CD177    ENSG00000204936:    1762 TTTCTGTACCCAGCCCTGGGTGCCTCTACCCAGACCTCCTGTTCATTCTCCCTTCCTCTT ENSG00000204936:    1821
                                  ||||||||||||||||||||||||||||||||||||||||| ||  ||| ||||||||||
CD177P1               19:43379360 TTTCTGTACCCAGCCCTGGGTGCCTCTACCCAGACCTCCTGATCTCTCTGCCTTCCTCTT               19:43379301

CD177    ENSG00000204936:    1822 TTATGGATTACACTTCT                                            ENSG00000204936:    1838
                                  | |||| ||||||
CD177P1               19:43379300 TCATGGGTTACAC----                                                           19:43379288

(Alternative 5′ donor of exon 4)
CD177    ENSG00000204936:    1839 TTATTTTCCCACATCCAGATCCCATGATGGGAGCTGCAGAAGGCCCCTTTGGGAAGGCTG ENSG00000204936:    1898
                                  ||||||||||||||||||||||||||||||||||||||||||||||||||||||||||||
CD177P1               19:43379287 TTATTTTCCCACATCCAGATCCCATGATGGGAGCTGCAGAAGGCCCCTTTGGGAAGGCTG               19:43379228

CD177    ENSG00000204936:    1899 AGCAGGTTGACTCTTGAGGCCAGCAAAAGTGGGGTGCAGAGAAGGTATCTGATCAAATCC ENSG00000204936:    1958
                                  ||||||||||||| ||||||||||||||||||||||||||||||||||||||||||||||
CD177P1               19:43379227 AGCAGGTTGACTCCTGAGGCCAGCAAAAGTGGGGTGCAGAGAAGGTATCTGATCAAATCC               19:43379168

CD177    ENSG00000204936:    1959 AGTGCCCTCTCAGTGCCCCCTCACTCTGTCTGTCACTTTCCTAGACCCAGGATCCTTGAG ENSG00000204936:    2018
                                  |||||| ||||||||||||||||||||||||||||||| ||||||| |||||||||||||
CD177P1               19:43379167 AGTGCCGTCTCAGTGCCCCCTCACTCTGTCTGTCACTTCCCTAGACGCAGGATCCTTGAG               19:43379108

CD177    ENSG00000204936:    2019 GTGCCCAGTCTGCTTGTCTATGGAAGGCTGTCTGGAGGGGACAACAGAAGAGATCTGCCC ENSG00000204936:    2078
                                  ||||||||||||||||||||||||||||||||||||||||||||||||||||||||||||
CD177P1               19:43379107 GTGCCCAGTCTGCTTGTCTATGGAAGGCTGTCTGGAGGGGACAACAGAAGAGATCTGCCC               19:43379048

CD177    ENSG00000204936:    2079 CAAGGGGACCACACACTGTTATGATGGCCTCCTCAGGCTCAGGGGAGGTAAGCCTGGGAC ENSG00000204936:    2138
                                  ||||||||||||||||||||||||||||||||||||||||||||||||||||||||||||
CD177P1               19:43379047 CAAGGGGACCACACACTGTTATGATGGCCTCCTCAGGCTCAGGGGAGGTAAGCCTGGGAC               19:43378988

CD177    ENSG00000204936:    2139 ATCGGGGTCCCTGTGGGGACTGAACTGGAAGGTCTGGGGACTGAGATCTTAGGCTTTGGG ENSG00000204936:    2198
                                  ||||||||||||| |||||||||||||||||||||||||||||||||||||||| |||||||
CD177P1               19:43378987 ATCGGGGTCCCTGCGGGGACTGAACTGGAAGGTCTGGGGACTGAGATCTTAGTCTTTGGG               19:43378928

CD177    ENSG00000204936:    2199 GAGTGA-                                                      ENSG00000204936:    2204
                                  ||||||
CD177P1               19:43378927 GAGTGAG                                                                     19:43378921

CD177    ENSG00000204936:    2205 GGTGAGCTGAGGCACGGCATGAGACCCAGAGGAGGGTGGGCCTGGCTTCCTGGAGCTATG ENSG00000204936:    2264
                                  ||||||||||||||||||||||||||||||||| ||||||||||||||||||||||||||
CD177P1               19:43378920 GGTGAGCTGAGGCACGGCATGAGACCCAGAGGATGGTGGGCCTGGCTTCCTGGAGCTATG               19:43378861

CD177    ENSG00000204936:    2265 CCTGCCTCTGAGGGTTGGGTGGTCCTGGAGGCAGCATCACTGACTCTCCCTCGCTCCCCC ENSG00000204936:    2324
                                  ||||||||||||||||||||||||||||||||||||||||||||||||||||||||||||
CD177P1               19:43378860 CCTGCCTCTGAGGGTTGGGTGGTCCTGGAGGCAGCATCACTGACTCTCCCTCGCTCCCCC               19:43378801

CD177    ENSG00000204936:    2325 TTTCTGCAGGAGGCATCTTCTCCAATCTGAGAGTCCAGGGATGCATGCCCCAGCCAGGTT ENSG00000204936:    2384
                                  ||||||||||||||||||||||||||||||||||||||||||||||||||||||||| ||
CD177P1               19:43378800 TTTCTGCAGGAGGCATCTTCTCCAATCTGAGAGTCCAGGGATGCATGCCCCAGCCAGTTT               19:43378741

CD177    ENSG00000204936:    2385 GCAACCTGCTCAATGGGACACAGGAAATTGGGCCCGTGGGTATGACTGAGAACTGCAATA ENSG00000204936:    2444
                                  |||||||||||||||||||||||||||||||||||||||||||||||||||||||| |||
CD177P1               19:43378740 GCAACCTGCTCAATGGGACACAGGAAATTGGGCCCGTGGGTATGACTGAGAACTGCGATA               19:43378681

CD177    ENSG00000204936:    2445 GGAAAGGTGAGTCCTGCCCCTAGGCTGTGCCCTGGACTGCAGCCTCGGGGCACGATGACA ENSG00000204936:    2504
                                   |||||||||||||||||||||||||||||||||||||||||||||||||||||||||||
CD177P1               19:43378680 TGAAAGGTGAGTCCTGCCCCTAGGCTGTGCCCTGGACTGCAGCCTCGGGGCACGATGACA               19:43378621

CD177    ENSG00000204936:    2505 CATGGGGCATCCAGAGCCATCACACCAAAGCCAGCAGGAGGAAGGTCAAGGGTGCAGGGT ENSG00000204936:    2564
                                  ||||||||||||||||||||||||||||||||||||||||||||||||||||||||||||
CD177P1               19:43378620 CATGGGGCATCCAGAGCCATCACACCAAAGCCAGCAGGAGGAAGGTCAAGGGTGCAGGGT               19:43378561

CD177    ENSG00000204936:    2565 GGGTTGCTTGGCATGTGGCTTTAGGCAACAGTGAGTGCTGGGGCCCCTGCTGGCTCTCAA ENSG00000204936:    2624
                                  ||||||||||||||||||||||||||||||||||||||||||||||||||||||||||||
CD177P1               19:43378560 GGGTTGCTTGGCATGTGGCTTTAGGCAACAGTGAGTGCTGGGGCCCCTGCTGGCTCTCAA               19:43378501

CD177    ENSG00000204936:    2625 GCTGCTGAGAGTGACTGGTTGCTTCAATTTCTGTGTCTCCACACCCCTGGCCTTGGGTAA ENSG00000204936:    2684
                                  ||||||||||||||||||||||||||||||||||||||||||||||||||||||||||||
CD177P1               19:43378500 GCTGCTGAGAGTGACTGGTTGCTTCAATTTCTGTGTCTCCACACCCCTGGCCTTGGGTAA               19:43378441

CD177    ENSG00000204936:    2685 CCCTAGGTCTCCCACTTCCCTAGGCCACCGTAGCCTCTCTCCCACCCGTATCAGCCCCCG ENSG00000204936:    2744
                                  ||||||||||||||||||||||||||||||||||||||||||||||||||||||||||||
CD177P1               19:43378440 CCCTAGGTCTCCCACTTCCCTAGGCCACCGTAGCCTCTCTCCCACCCGTATCAGCCCCCG               19:43378381

CD177    ENSG00000204936:    2745 CCCCGCTCATGGTGCTGGAGTGCCCCAGCAGCCTTGCCTGCACAGGAGAGAACATCAGAG ENSG00000204936:    2804
                                  ||||||||||||||||||||||||||||||||||||||||||||||||||||||||||||
CD177P1               19:43378380 CCCCGCTCATGGTGCTGGAGTGCCCCAGCAGCCTTGCCTGCACAGGAGAGAACATCAGAG               19:43378321

CD177    ENSG00000204936:    2805 AGAAAATCAAGGGGACTTTGGGTGTGACAGAGAAAGCAAGAGTTAAAGGAGGACACTGAG ENSG00000204936:    2864
                                  ||||||||||||||||||||||||||||||||||||||||||||||||||||||||||||
CD177P1               19:43378320 AGAAAATCAAGGGGACTTTGGGTGTGACAGAGAAAGCAAGAGTTAAAGGAGGACACTGAG               19:43378261

CD177    ENSG00000204936:    2865 AGGGTCCCCACTTACCCATTTAGGTGCTGCCATGGAAGAGAGTGCCAGGGAATGTCTCCA ENSG00000204936:    2924
                                  ||||||||||||||||||||||||||||||||||||||||||||||||||||||||||||
CD177P1               19:43378260 AGGGTCCCCACTTACCCATTTAGGTGCTGCCATGGAAGAGAGTGCCAGGGAATGTCTCCA               19:43378201

CD177    ENSG00000204936:    2925 TAAGAAGGGCTCCAAAGCAACAGGAAGCCATGGTGGGGAGGTGGGGGTCTTATAGGAATC ENSG00000204936:    2984
                                  ||||||||||||||||||||||||||||||||||||||||||||||||||||||||||||
CD177P1               19:43378200 TAAGAAGGGCTCCAAAGCAACAGGAAGCCATGGTGGGGAGGTGGGGGTCTTATAGGAATC               19:43378141

CD177    ENSG00000204936:    2985 CCAGCTTCCCTCCTAGAAGCAAAGTCTTCCCTGCCCCTGCCTCAGTTTCCTTGTCTGCCC ENSG00000204936:    3044
                                  ||||||||||||||||||||||||||||||||||||||||||||||||||||||||||||
CD177P1               19:43378140 CCAGCTTCCCTCCTAGAAGCAAAGTCTTCCCTGCCCCTGCCTCAGTTTCCTTGTCTGCCC               19:43378081

CD177    ENSG00000204936:    3045 ACATCACGCTAACCCTTAGAGCTTAGAAGAGAGAGTTGTGTGAGCTCTGCACACATTCCT ENSG00000204936:    3104
                                  ||||||||||||||||||||||||||||||||||||||||||||||||||||||||||||
CD177P1               19:43378080 ACATCACGCTAACCCTTAGAGCTTAGAAGAGAGAGTTGTGTGAGCTCTGCACACATTCCT               19:43378021

CD177    ENSG00000204936:    3105 CTGTGTATTTGGCTGTTTCTCCCTAGCTAGGCTGGGAGCCTGAGGCTAGGGGCTTGGTAC ENSG00000204936:    3164
                                  ||||||||||||||||||||||||||||||||||||||||||||||||||||||||||||
CD177P1               19:43378020 CTGTGTATTTGGCTGTTTCTCCCTAGCTAGGCTGGGAGCCTGAGGCTAGGGGCTTGGTAC               19:43377961
```

```
CD177    ENSG00000204936:    3165 CACTGGCACCAGGGTTTCCAGCCTTGGGTAGCTCAGGAGACGGCTCAGGAAGGGTACCAG ENSG00000204936:    3224
                                  ||||||||||||||||||||||||||||||||||||||||||||||||||||||||||||
CD177P1             19:43377960 CACTGGCACCAGGGTTTCCAGCCTTGGGTAGCTCAGGAGACGGCTCAGGAAGGGTACCAG             19:43377901

CD177    ENSG00000204936:    3225 GAGGTGTTTGCTCAAGTGAGGACATGCAGGGCTCACTTGAGCAAAGCTCCCTCATGAGTC ENSG00000204936:    3284
                                  ||||||||||||||||||||||||||||||||||||||||||||||||||||||||||||
CD177P1             19:43377900 GAGGTGTTTGCTCAAGTGAGGACATGCAGGGCTCACTTGAGCAAAGCTCCCTCATGAGTC             19:43377841

CD177    ENSG00000204936:    3285 AGACCCTGTTTCTACCTGAAGGGCCTCCAGGACTAGGAGGGGGAGGTGCTACAGACACAG ENSG00000204936:    3344
                                  ||||||||||||||||||||||||||||||||||||||||||||||||||||||||||||
CD177P1             19:43377840 AGACCCTGTTTCTACCTGAAGGGCCTCCAGGACTAGGAGGGGGAGGTGCTACAGACACAG             19:43377781

CD177    ENSG00000204936:    3345 ATGGTCACAGCTTGAGGTGATCAGGGCTGTGGCAAACGGGGATGTGGGGCCTGGGGCAGC ENSG00000204936:    3404
                                  ||||||||||||||||||||||||||||||||||||||||||||||||||||||||||||
CD177P1             19:43377780 ATGGTCACAGCTTGAGGTGATCAGGGCTGTGGCAAACGGGGATGTGGGGCCTGGGGCAGC             19:43377721

CD177    ENSG00000204936:    3405 TCAGAGGAGGAAAATACTGATGTTGTTGGGGTAGAGAGTCAAGGAGGGCTTTACAGAGGA ENSG00000204936:    3464
                                  ||||||||||||||||||||||||||||||||||||||||||||||||||||||||||||
CD177P1             19:43377720 TCAGAGGAGGAAAATACTGATGTTGTTGGGGTAGAGAGTCAAGGAGGGCTTTACAGAGGA             19:43377661

CD177    ENSG00000204936:    3465 GGTGGCCTGTGAACCGTGTGTTAAAGATGACCTACAGCTTATTGGTAGAAGAGGAAGAGG ENSG00000204936:    3524
                                  ||||||||||||||||||||||||||||||||||||||||||||||||||||||||||||
CD177P1             19:43377660 GGTGGCCTGTGAACCGTGTGTTAAAGATGACCTACAGCTTATTGGTAGAAGAGGAAGAGG             19:43377601

CD177    ENSG00000204936:    3525 ACATTGGTGGCAGAGGGAACAGTGTGTAGCAGCTGCATAGGCCTGGAGGTGGGAGTGAGC ENSG00000204936:    3584
                                  ||||||||||||||||||||||||||||||||||||||||||||||||||||||||||||
CD177P1             19:43377600 ACATTGGTGGCAGAGGGAACAGTGTGTAGCAGCTGCATAGGCCTGGAGGTGGGAGTGAGC             19:43377541

CD177    ENSG00000204936:    3585 CTGCTGTTTTCAAGAAGAGTCCCTCACTCCACCTGAGAAGGACTCATCAAGATGAGGGGG ENSG00000204936:    3644
                                  ||||||||||||||||||||||||||||||||||||||||||||||||||||||||||||
CD177P1             19:43377540 CTGCTGTTTTCAAGAAGAGTCCCTCACTCCACCTGAGAAGGACTCATCAAGATGAGGGGG             19:43377481

CD177    ENSG00000204936:    3645 TACAGCTGGGATTGGGGGTGATTCGAAGTTTTCCAGGTGGACAAGGTGGGTGTGGGGTCG ENSG00000204936:    3704
                                  ||||||||||||||||||||||||||||||||||||||||||||||||||||||||||||
CD177P1             19:43377480 TACAGCTGGGATTGGGGGTGATTCGAAGTTTTCCAGGTGGACAAGGTGGGTGTGGGGTCG             19:43377421

CD177    ENSG00000204936:    3705 AAGAGGGAGCTGCTCCAAGCCAAAGGCACAGCAGGTGTGAGGCTGAGGAGGAAATGAACT ENSG00000204936:    3764
                                  ||||||||||||||||||||||||||||||||||||||||||||||||||||||||||||
CD177P1             19:43377420 AAGAGGGAGCTGCTCCAAGCCAAAGGCACAGCAGGTGTGAGGCTGAGGAGGAAATGAACT             19:43377361

CD177    ENSG00000204936:    3765 GGCATGCCTTCAGAATGGATACTGGTGGGGGAGGAAGAGTTAAGTGGGGAACCTGGTGGT ENSG00000204936:    3824
                                  ||||||||||||||||||||||||||||||||||||||||||||||||||||||||||||
CD177P1             19:43377360 GGCATGCCTTCAGAATGGATACTGGTGGGGGAGGAAGAGTTAAGTGGGGAACCTGGTGGT             19:43377301

CD177    ENSG00000204936:    3825 CGCTCAAGTACCTGAAAACCCAGGCTGAGGGGTGGACCCTCTCCTGAAGGCAGGGGAGCC ENSG00000204936:    3884
                                  ||||||||||||||||||||||||||||||||||||||||||||||||||||||||||||
CD177P1             19:43377300 CGCTCAAGTACCTGAAAACCCAGGCTGAGGGGTGGACCCTCTCCTGAAGGCAGGGGAGCC             19:43377241

CD177    ENSG00000204936:    3885 ATGGAAGATTGTGGAGCAGGGAGGGCAGGGTCTGAGCCATTTATCTTTCCTTTTGACTGA ENSG00000204936:    3944
                                  ||||||||||||||||||||||||||||||||||||||||||||||||||||||||||||
CD177P1             19:43377240 ATGGAAGATTGTGGAGCAGGGAGGGCAGGGTCTGAGCCATTTATCTTTCCTTTTGACTGA             19:43377181

CD177    ENSG00000204936:    3945 TGCTGCTGTTAAGGATCTGGCCCTGGTCTCTGGGACCAGATGACACTGATAACCCAGAAG ENSG00000204936:    4004
                                  ||||||||||||||||||||||||||||||||||||||||||||||||||||||||||||
CD177P1             19:43377180 TGCTGCTGTTAAGGATCTGGCCCTGGTCTCTGGGACCAGATGACACTGATAACCCAGAAG             19:43377121

CD177    ENSG00000204936:    4005 GTATTTCAGAAACAAATCAGACGCAACACATGCCACACACACAGCCCACAATAAGCACAG ENSG00000204936:    4064
                                  ||||||||||||||||||||||||||||||||||||||||||||||||||||||||||||
CD177P1             19:43377120 GTATTTCAGAAACAAATCAGACGCAACACATGCCACACACACAGCCCACAATAAGCACAG             19:43377061

CD177    ENSG00000204936:    4065 CAGACACGAATGCGTCACACCATGACACATACACACCACACATCACATACCACACACACA ENSG00000204936:    4124
                                  ||||||||||||||||||||||||||||||||||||||||||||||||||||||||||||
CD177P1             19:43377060 CAGACACGAATGCGTCACACCATGACACATACACACCACACATCACATACCACACACACA             19:43377001

CD177    ENSG00000204936:    4125 CTCCCCCATACCCCTGACATACCACCTATGGAACACAAATCACACACAGAGCACACAATA ENSG00000204936:    4184
                                  ||||||||||||||||||||||||||||||||||||||||||||||||||||||||||||
CD177P1             19:43377000 CTCCCCCATACCCCTGACATACCACCTATGGAACACAAATCACACACAGAGCACACAATA             19:43376941

CD177    ENSG00000204936:    4185 TGCACAACACACACAAATGCATCACACGAGTGCACAATTACTACGTGTACATGTAGTGTG ENSG00000204936:    4244
                                  ||||||||||||||||||||||||||||||||||||||||||||||||||||||||||||
CD177P1             19:43376940 TGCACAACACACACAAATGCATCACACGAGTGCACAATTACTACGTGTACATGTAGTGTG             19:43376881

CD177    ENSG00000204936:    4245 TATGCCACATACTACACGTCAGGTACCACACACACTCTCCCTCTCAACATATCAGACATG ENSG00000204936:    4304
                                  ||||||||||||||||||||||||||||||||||||||||||||||||||||||||||||
CD177P1             19:43376880 TATGCCACATACTACACGTCAGGTACCACACACACTCTCCCTCTCAACATATCAGACATG             19:43376821

CD177    ENSG00000204936:    4305 CAATACATACCACACACAACACACAATACACACAACACATCTGGCATGCCACACACACCA ENSG00000204936:    4364
                                  ||||||||||||||||||||||||||||||||||||||||||||||||||||||||||||
CD177P1             19:43376820 CAATACATACCACACACAACACACAATACACACAACACATCTGGCATGCCACACACACCA             19:43376761

CD177    ENSG00000204936:    4365 CATGCAAGCAAAACACAGATACACAGAACACACACCACACCAAACACACAAAACAAACAC ENSG00000204936:    4424
                                  ||||||||||||||||||||||||||||||||||||||||||||||||||||||||||||
CD177P1             19:43376760 CATGCAAGCAAAACACAGATACACAGAACACACACCACACCAAACACACAAAACAAACAC             19:43376701

CD177    ENSG00000204936:    4425 ATACCACATATATCACACACAGTATGTCTACACATGCACGCACCACACAAACATACCATG ENSG00000204936:    4484
                                  ||||||||||||||||||||||||||||||||||||||||||||||||||||||||||||
CD177P1             19:43376700 ATACCACATATATCACACACAGTATGTCTACACATGCACGCACCACACAAACATACCATG             19:43376641

CD177    ENSG00000204936:    4485 AACACACCACACACCCCATACAACCACACCCCCCACATGTATACTACGCACTCACACACA ENSG00000204936:    4544
                                  ||||||||||||||||||||||||||||||||||||||||||||||||||||||||||||
CD177P1             19:43376640 AACACACCACACACCCCATACAACCACACCCCCCACATGTATACTACGCACTCACACACA             19:43376581

CD177    ENSG00000204936:    4545 GCACACATACCTGCACACATCACGCATGTGCACACACTACACAAACGCACCACATAAAAC ENSG00000204936:    4604
                                  ||||||||||||||||||||||||||||||||||||||||||||||||||||||||||||
CD177P1             19:43376580 GCACACATACCTGCACACATCACGCATGTGCACACACTACACAAACGCACCACATAAAAC             19:43376521

CD177    ENSG00000204936:    4605 ACAGCGACTATGCACCAATCAGTGCTGTTCATAGAGATTCA---                 ENSG00000204936:    4645
                                  ||||||||||||||||||||||||||||||||||||||||||||
CD177P1             19:43376520 ACAGCGACTATGCACCAATCAGTGCTGTTCATAGAGATTCATCT                             19:43376477

CD177    ENSG00000204936:    4646 TCTGCTTTCAACACTGGAGCCAGATCTTGCAGGCTGTTTTGCCTCCCTGGTGAAGTGCAG ENSG00000204936:    4705
                                  ||||||||||||||||||||||||||||||||||||||||||||||||||||||||||||
```

```
CD177P1                 19:43376476 TCTGCTTTCAACACTGGAGCCAGATCTTGCAGGCTGTTTTGCCTCCCTGGTGAAGTGCAG                 19:43376417

CD177    ENSG00000204936:      4706 TTCAGCAGTTTAGCAGACAGTGAGCATCACAAGACCAGGCATCAGAAACTGGGGGAACAA ENSG00000204936:     4765
                                    ||||||||||||||||||||||||||||||||||||||||||||||||||||||||||||
CD177P1                 19:43376416 TTCAGCAGTTTAGCAGACAGTGAGCATCACAAGACCAGGCATCAGAAACTGGGGGAACAA                 19:43376357

CD177    ENSG00000204936:      4766 CAAGACGTTGGGGACCAAGTGCAGGAGAGAAGAGGCTTCAGGGTGATGGAACCGCTTATA ENSG00000204936:     4825
                                    ||||||||||||||||||||||||||||||||||||||||||||||||||||||||||||
CD177P1                 19:43376356 CAAGACGTTGGGGACCAAGTGCAGGAGAGAAGAGGCTTCAGGGTGATGGAACCGCTTATA                 19:43376297

CD177    ENSG00000204936:      4826 AGAAGCCGCAGCAGTGGGAGGGCTGGGTGCAAGGATGCAGGTTACTAAAAGGGGCCATGG ENSG00000204936:     4885
                                    ||||||||||||||||||||||||||||||||||||||||||||||||||||||||||||
CD177P1                 19:43376296 AGAAGCCGCAGCAGTGGGAGGGCTGGGTGCAAGGATGCAGGTTACTAAAAGGGGCCATGG                 19:43376237

CD177    ENSG00000204936:      4886 CAGCAGCCCCTTGTGAAGAACCAAGGGGCCCGGGCAGATAAGGCCCTGGCTGCCCCTAGA ENSG00000204936:     4945
                                    ||||||||||||||||||||||||||||||||||||||||||||||||||||||||||||
CD177P1                 19:43376236 CAGCAGCCCCTTGTGAAGAACCAAGGGGCCCGGGCAGATAAGGCCCTGGCTGCCCCTAGA                 19:43376177

CD177    ENSG00000204936:      4946 GGAGCTGGGCAGCGGTCCCAGTGAGAGAGCAGGGAAGAGCCCACGTCTGAATCAGGGACA ENSG00000204936:     5005
                                    ||||||||||||||||||||||||||||||||||||||||||||||||||||||||||||
CD177P1                 19:43376176 GGAGCTGGGCAGCGGTCCCAGTGAGAGAGCAGGGAAGAGCCCACGTCTGAATCAGGGACA                 19:43376117

CD177    ENSG00000204936:      5006 CGTGGGTCCCAGGCCCAGCTCTGCCGCCGAAACAGCTTTCCAGCCTCAGCTTCACACGAG ENSG00000204936:     5065
                                    ||||||||||||||||||||||||||||||||||||||||||||||||||||||||||||
CD177P1                 19:43376116 CGTGGGTCCCAGGCCCAGCTCTGCCGCCGAAACAGCTTTCCAGCCTCAGCTTCACACGAG                 19:43376057

CD177    ENSG00000204936:      5066 GATCAAAAGTCTCCATATCTCATGGAGCCTCTTTCCCTGGATTTGGGGAAGAGGAGGGGA ENSG00000204936:     5125
                                    ||||||||||||||||||||||||||||||||||||||||||||||||||||||||||||
CD177P1                 19:43376056 GATCAAAAGTCTCCATATCTCATGGAGCCTCTTTCCCTGGATTTGGGGAAGAGGAGGGGA                 19:43375997

CD177    ENSG00000204936:      5126 TGGTTGCAGACATGGGACGGAAGCAGATGCAAAGACCATGATGTGGGTGAGTGCCTGCCA ENSG00000204936:     5185
                                    ||||||||||||||||||||||||||||||||||||||||||||||||||||||||||||
CD177P1                 19:43375996 TGGTTGCAGACATGGGACGGAAGCAGATGCAAAGACCATGATGTGGGTGAGTGCCTGCCA                 19:43375937

CD177    ENSG00000204936:      5186 GAAACACCTGTGCGGAGCAGAGTGATGGAGGGAGCAGGGAGGCGGCCGCGTCTTATCAGG ENSG00000204936:     5245
                                    ||||||||||||||||||||||||||||||||||||||||||||||||||||||||||||
CD177P1                 19:43375936 GAAACACCTGTGCGGAGCAGAGTGATGGAGGGAGCAGGGAGGCGGCCGCGTCTTATCAGG                 19:43375877

CD177    ENSG00000204936:      5246 CCTCCCGGGCCATTGGAAGGGCTTAGAATTTTATTCTCCAGAATGGGGACCTCCTGGAGT ENSG00000204936:     5305
                                    ||||||||||||||||||||||||||||||||||||||||||||||||||||||||||||
CD177P1                 19:43375876 CCTCCCGGGCCATTGGAAGGGCTTAGAATTTTATTCTCCAGAATGGGGACCTCCTGGAGT                 19:43375817

CD177    ENSG00000204936:      5306 CTTCGGAGCAGAGGAGTGACATGAACTGACTTAGGTTTACCTAGCCTCCCTCTGCCTGCT ENSG00000204936:     5365
                                    ||||||||||||||||||||||||||||||||||||||||||||||||||||||||||||
CD177P1                 19:43375816 CTTCGGAGCAGAGGAGTGACATGAACTGACTTAGGTTTACCTAGCCTCCCTCTGCCTGCT                 19:43375757

CD177    ENSG00000204936:      5366 GGGTGGAGAATGGAGCGAGAGGAACAGTGACAGGCGGGTGCAGGGATTGCCCCAGGAGTC ENSG00000204936:     5425
                                    ||||||||||||||||||||||||||||||||||||||||||||||||||||||||||||
CD177P1                 19:43375756 GGGTGGAGAATGGAGCGAGAGGAACAGTGACAGGCGGGTGCAGGGATTGCCCCAGGAGTC                 19:43375697

CD177    ENSG00000204936:      5426 AGGTGGGAGTGAAGCGGGGGGCTTGGAGGCAGCAGAGGTGGTGACGTGGGGTTGGGTTTT ENSG00000204936:     5485
                                    ||||||||||||||||||||||||||||||||||||||||||||||||||||||||||||
CD177P1                 19:43375696 AGGTGGGAGTGAAGCGGGGGGCTTGGAGGCAGCAGAGGTGGTGACGTGGGGTTGGGTTTT                 19:43375637

CD177    ENSG00000204936:      5486 GAAGATTTTCTGCAGCTCAAGCCAGTAGGATTACTTGAGGAACACAATGTGGGAGAGAAA ENSG00000204936:     5545
                                    ||||||||||||||||||||||||||||||||||||||||||||||||||||||||||||
CD177P1                 19:43375636 GAAGATTTTCTGCAGCTCAAGCCAGTAGGATTACTTGAGGAACACAATGTGGGAGAGAAA                 19:43375577

CD177    ENSG00000204936:      5546 GAGCGTGGTCAAGGACAACACCAATGTGTTCAGCTAGACTGGCAGAAACGAGCCGCCACG ENSG00000204936:     5605
                                    ||||||||||||||||||||||||||||||||||||||||||||||||||||||||||||
CD177P1                 19:43375576 GAGCGTGGTCAAGGACAACACCAATGTGTTCAGCTAGACTGGCAGAAACGAGCCGCCACG                 19:43375517

CD177    ENSG00000204936:      5606 GGTGTAAGTAGGGGAGATGGGAGTGGGGAGAGAAGGGTGGGAGAAGGGAGCAGGAGCCAT ENSG00000204936:     5665
                                    ||||||||||||||||||||||||||||||||||||||||||||||||||||||||||||
CD177P1                 19:43375516 GGTGTAAGTAGGGGAGATGGGAGTGGGGAGAGAAGGGTGGGAGAAGGGAGCAGGAGCCAT                 19:43375457

CD177    ENSG00000204936:      5666 GATTTTGATTTTGTCATCTGTGAAGTATGCAAATGAGCAGCCCTCCAGGGTGGCATCGGT ENSG00000204936:     5725
                                    ||||||||||||||||||||||||||||||||||||||||||||||||||||||||||||
CD177P1                 19:43375456 GATTTTGATTTTGTCATCTGTGAAGTATGCAAATGAGCAGCCCTCCAGGGTGGCATCGGT                 19:43375397

CD177    ENSG00000204936:      5726 TCTGCAGGTCGCATGTGTTGGTGGAAGCTGGGGGCTCAGTCTAAGAGTCCCTGCATCATG ENSG00000204936:     5785
                                    ||||||||||||||||||||||||||||||||||||||||||||||||||||||||||||
CD177P1                 19:43375396 TCTGCAGGTCGCATGTGTTGGTGGAAGCTGGGGGCTCAGTCTAAGAGTCCCTGCATCATG                 19:43375337

CD177    ENSG00000204936:      5786 GGACCGATCCCCCCTGCTCTGTGATGGCCGTGAGGGGAGGCCAGGGGCTGTGGTTTCACA ENSG00000204936:     5845
                                    ||||||||||||||||||||||||||||||||||||||||||||||||||||||||||||
CD177P1                 19:43375336 GGACCGATCCCCCCTGCTCTGTGATGGCCGTGAGGGGAGGCCAGGGGCTGTGGTTTCACA                 19:43375277

CD177    ENSG00000204936:      5846 CTAGAGCCTTGAGGGCCTCGGGGCCAAGGTAGCCGAGGATGGTCTTCAGTACCTGTCTCC ENSG00000204936:     5905
                                    ||||||||||||||||||||||||||||||||||||||||||||||||||||||||||||
CD177P1                 19:43375276 CTAGAGCCTTGAGGGCCTCGGGGCCAAGGTAGCCGAGGATGGTCTTCAGTACCTGTCTCC                 19:43375217

CD177    ENSG00000204936:      5906 CTCCTGCTCCCAACCCCGACCCTGAGGGTCCAGAGTCCCTGGGTTCCCAGCCTACCTCTG ENSG00000204936:     5965
                                    ||||||||||||||||||||||||||||||||||||||||||||||||||||||||||||
CD177P1                 19:43375216 CTCCTGCTCCCAACCCCGACCCTGAGGGTCCAGAGTCCCTGGGTTCCCAGCCTACCTCTG                 19:43375157

CD177    ENSG00000204936:      5966 CCACTCACAAGCTGTGTGGCATGGGCAGCTACTGAGCAATCCTGCCTCTGTTTCCCTTGT ENSG00000204936:     6025
                                    ||||||||||||||||||||||||||||||||||||||||||||||||||||||||||||
CD177P1                 19:43375156 CCACTCACAAGCTGTGTGGCATGGGCAGCTACTGAGCAATCCTGCCTCTGTTTCCCTTGT                 19:43375097

CD177    ENSG00000204936:      6026 TGCAAATGAGGGTGTTGATTGTGATCACACCACCTACTGCTGGGGTGCTGTGGGGAAATG ENSG00000204936:     6085
                                    ||||||||||||||||||||||||||||||||||||||||||||||||||||||||||||
CD177P1                 19:43375096 TGCAAATGAGGGTGTTGATTGTGATCACACCACCTACTGCTGGGGTGCTGTGGGGAAATG                 19:43375037

CD177    ENSG00000204936:      6086 AGGCATCCCAGATAAGCTGTGTTCCCTGGAACCTTAGATGGGTGGATCTGTGGGAAGCAA ENSG00000204936:     6145
                                    ||||||||||||||||||||||||||||||||||||||||||||||||||||||||||||
CD177P1                 19:43375036 AGGCATCCCAGATAAGCTGTGTTCCCTGGAACCTTAGATGGGTGGATCTGTGGGAAGCAA                 19:43374977

CD177    ENSG00000204936:      6146 TGAGGTCAGCCAGGTGCAGAGGCAGTGCTCAGCGGCAGTGCTCAGCCCACCCTGGCCCTT ENSG00000204936:     6205
                                    ||||||||||||||||||||||||||||||||||||||||||||||||||||||||||||
CD177P1                 19:43374976 TGAGGTCAGCCAGGTGCAGAGGCAGTGCTCAGCGGCAGTGCTCAGCCCACCCTGGCCCTT                 19:43374917
```

```
CD177    ENSG00000204936:     6206 CGCATCTTGTTTGGAACATAGGGCTTTGCAACTGAAAGGTATGAGAAAGGTCTCAGGCTG ENSG00000204936:     6265
                                   ||||||||||||||||||||||||||||||||||||||||||||||||||||||||||||
CD177P1                19:43374916 CGCATCTTGTTTGGAACATAGGGCTTTGCAACTGAAAGGTATGAGAAAGGTCTCAGGCTG                19:43374857

CD177    ENSG00000204936:     6266 CCTTCCTTATTATTGAAATGAGGACCTTAAAGCTCAGATGGGGAAGCAGAGATGGGATGA ENSG00000204936:     6325
                                   ||||||||||||||||||||||||||||||||||||||||||||||||||||||||  ||
CD177P1                19:43374856 CCTTCCTTATTATTGAAATGAGGACCTTAAAGCTCAGATGGGGAAGCAGAGATGGGCAGA                19:43374797

CD177    ENSG00000204936:     6326 GGATAAGGAAGAGCTTCCATTGCTCTCACAGCTAAAGCAAGGAACGGCCGTTCCCCCAGG ENSG00000204936:     6385
                                   || | ||||||||||||||||||||||||||| |||||||||| ||| |||| | ||||
CD177P1                19:43374796 GGCTCAGGAAGAGCTTCCATTGCTCTCACAGCTGAAGCAAGGAATGGCTGTTCTCACAGG                19:43374737

CD177    ENSG00000204936:     6386 GCTGTGAGAGGACGTCCCTGCGGGTGTTCAAGCAGAAACAGGGAACAATGGGGGTGGGAT ENSG00000204936:     6445
                                   |||||||||||||||||||||||||||||||||||||||||||||||||||||||||| |
CD177P1                19:43374736 GCTGTGAGAGGACGTCCCTGCGGGTGTTCAAGCAGAAACAGGGAACAATGGGGGTGGGGT                19:43374677

CD177    ENSG00000204936:     6446 TTCGACTCCCAAGTCCCTTCTGAATCCTTGGTTAAGGGAATCTGTGGCCAGGTCACCTGG ENSG00000204936:     6505
                                   || ||||||||||||||||||||||| |||||| ||||| || ||||||||||||||||
CD177P1                19:43374676 TTGGACTCCCAAGTCCCTTCTGAATCCGTGGTTGAGGGACTCCGTGGCCAGGTCACCTGG                19:43374617

CD177    ENSG00000204936:     6506 AGTGTGACTCAAGAGTGTGATCACCTTCCCTAGCCCAGGACGTGGAGGGACAGACATGGT ENSG00000204936:     6565
                                   |||||||||||||||  |||||||||||||||||||||||||||||||||||||||||||
CD177P1                19:43374616 AGTGTGACTCAAGAGCATGATCACCTTCCCTAGCCCAGGACGTGGAGGGACAGACATGGT                19:43374557

CD177    ENSG00000204936:     6566 GGGTTCCTGGCTTACACACACCCTGGGATTCCTCTCCCCAGATTTTCTGACCTGTCATCG ENSG00000204936:     6625
                                   |||||||||||||||||| |||||||| |||||||||||||||| ||||||||||||||||
CD177P1                19:43374556 GGGTTCCTGGCTTACACAGACCCTGGGTTTCCTCTCCCCAGATGTTCTGACCTGTCATCG                19:43374497

CD177    ENSG00000204936:     6626 GGGGACCACCATTATGACACACGGAAACTTGGCTCAAGAACCCACTGATTGGACCACATC ENSG00000204936:     6685
                                   |||||||||| | | ||  || | |||||||  | |||||||||||||||| |||||||
CD177P1                19:43374496 GGGGACCACCTTGAAGAAGCAGGAAAACTTGAGTAAAGAACCCACTGATTGGGCCACATC                19:43374437

CD177    ENSG00000204936:     6686 GAATACCGAGATGTGCGAGGTGGGGCAGGTGTGTCAGGAGACGCTGCTGCTCCTAGATGT ENSG00000204936:     6745
                                    |||||||||| |||||||||||||||||||||||||||||| ||||||||||| |||||||
CD177P1                19:43374436 TAATACCGAGACGTGCGAGGTGGGGCAGGTGTGTCAGGAGATGCTGCTGCTCATAGATGT                19:43374377

CD177    ENSG00000204936:     6746 AGGTACGTGGACTGAGGTAGAAGACGAACACCTGTCCCAAGTCCCTGGCAGCTCCCTCCA ENSG00000204936:     6805
                                   ||||||||||||||||||||||||||||||||||||||||||||||||||||||||||||
CD177P1                19:43374376 AGGTACGTGGACTGAGGTAGAAGACGAACACCTGTCCCAAGTCCCTGGCAGCTCCCTCCA                19:43374317

CD177    ENSG00000204936:     6806 CCCCACTGGATTCTTTCCCCTGAATTTCCTGCTCAGCTCCCTTCACATCTGCAATTTTCA ENSG00000204936:     6865
                                   ||||||||||||||||||||||||||||||||||||||||||||||||||||||||||||
CD177P1                19:43374316 CCCCACTGGATTCTTTCCCCTGAATTTCCTGCTCAGCTCCCTTCACATCTGCAATTTTCA                19:43374257

CD177    ENSG00000204936:     6866 ATCCGACTCTTCTTCTCTCTGCATCTTGGGTTCCCCTTATGAAAACTGGGGGTGAAGCAG ENSG00000204936:     6925
                                   ||||||||||||||||||||||||||||||||||||||||||||||||||||||||||||
CD177P1                19:43374256 ATCCGACTCTTCTTCTCTCTGCATCTTGGGTTCCCCTTATGAAAACTGGGGGTGAAGCAG                19:43374197

CD177    ENSG00000204936:     6926 TGGGAATGACAGTGTCTGCCTTTCTGGGGAGGGGTGACCACGTATGCGGTAATCCCTGCC ENSG00000204936:     6985
                                   ||||||||||||||||||||||||||||||||||||||||||||||||||||||||||||
CD177P1                19:43374196 TGGGAATGACAGTGTCTGCCTTTCTGGGGAGGGGTGACCACGTATGCGGTAATCCCTGCC                19:43374137

CD177    ENSG00000204936:     6986 CAGGGTGGAGAACCGTGCCAACACCGAGGACTCTCAGTGGCTTCATACATTCTGGAAATG ENSG00000204936:     7045
                                   ||||||||||||||||||||||||||||||||||||||||||||||||||||||||||||
CD177P1                19:43374136 CAGGGTGGAGAACCGTGCCAACACCGAGGACTCTCAGTGGCTTCATACATTCTGGAAATG                19:43374077

CD177    ENSG00000204936:     7046 TTTATCCAGCATTTGTTTTAGGTGCCGCAGATCCAATCCCATCTGTATTCCTGCAGCTTT ENSG00000204936:     7105
                                   ||||||||||||||||||||||||||||||||||||||||||||||||||||||||||||
CD177P1                19:43374076 TTTATCCAGCATTTGTTTTAGGTGCCGCAGATCCAATCCCATCTGTATTCCTGCAGCTTT                19:43374017

CD177    ENSG00000204936:     7106 ATATGCCTTGAGAAACAGGAAACAAACAAACAAAAAGCAGCAAAGTATGGGGAATGGAGA ENSG00000204936:     7165
                                   ||||||||||||||||||||||||||||||||||||||||||||||||||||||||||||
CD177P1                19:43374016 ATATGCCTTGAGAAACAGGAAACAAACAAACAAAAAGCAGCAAAGTATGGGGAATGGAGA                19:43373957

CD177    ENSG00000204936:     7166 TCCCAGCTCTGCAGAGAAATCTGGCAAGGAAAAGGAGATAGGAAAAGTGAGGGGAAGGCT ENSG00000204936:     7225
                                   ||||||||||| ||||||||||||||||||||||||||||||||||||||||||||||||
CD177P1                19:43373956 TCCCAGCTCTGGAGAGAAATCTGGCAAGGAAAAGGAGATAGGAAAAGTGAGGGGAAGGCT                19:43373897

CD177    ENSG00000204936:     7226 GCAATTTTAAGCTCAGTGCTCAGGGGAGACTCATGGAGAAGGTGACCTTTGAGCGAGAAC ENSG00000204936:     7285
                                   |||||||||||||||||||||||||||||||||||||||||||||| |||||||||||||
CD177P1                19:43373896 GCAATTTTAAGCTCAGTGCTCAGGGGAGACTCATGGAGAAGGTGACGTTTGAGCGAGAAC                19:43373837

CD177    ENSG00000204936:     7286 TGGGAGGAGGCAGAGCTTCGGGCAGGACCCAGGCCCCAGCCTCCGTCTTTGCCCCACACT ENSG00000204936:     7345
                                   ||||||||||||||||||||||||||||||||||||||||||||||||||||||||||||
CD177P1                19:43373836 TGGGAGGAGGCAGAGCTTCGGGCAGGACCCAGGCCCCAGCCTCCGTCTTTGCCCCACACT                19:43373777

CD177    ENSG00000204936:     7346 AAACATGACCCAGCAGTTGTGATCAGGGCATTCACCCTCTGCCTGGGGGGTATTGTGAAG ENSG00000204936:     7405
                                   ||||||||||||||||||||||||||||||||||||||||||||||||||||||||||||
CD177P1                19:43373776 AAACATGACCCAGCAGTTGTGATCAGGGCATTCACCCTCTGCCTGGGGGGTATTGTGAAG                19:43373717

CD177    ENSG00000204936:     7406 GGCAGGGAGTCCAGCTCTGGAGCCCCTGCTGCCCTGGGAGCTGCCCAGTCCCCAGCCCAG ENSG00000204936:     7465
                                   ||||||||||||||||||||||||||||||||||||||||||||||||||||||||||||
CD177P1                19:43373716 GGCAGGGAGTCCAGCTCTGGAGCCCCTGCTGCCCTGGGAGCTGCCCAGTCCCCAGCCCAG                19:43373657

CD177    ENSG00000204936:     7466 CTTTCCCTCTCACCCTCAGGACTCACATCAACCCTGGTGGGGACAAAAGGCTGCAGCACT ENSG00000204936:     7525
                                   |||||||||||||||||||||||||||||||||||||||| |||  || |||||||| ||
CD177P1                19:43373656 CTTTCCCTCTCACCCTCAGGACTCACATCAACCCTGGTGGCGACCTAAAGCTGCAGCGCT                19:43373597

CD177    ENSG00000204936:     7526 GTTGGGGCTCAAAATTCCCAGAAGACCACCATCCACTCAGCCCCTCCTGGGGTGCTTGTG ENSG00000204936:     7585
                                   ||||||||||||||||||||||||||||||||||||||||||||||||||||||||||||
CD177P1                19:43373596 GTTGGGGCTCAAAATTCCCAGAAGACCACCATCCACTCAGCCCCTCCTGGGGTGCTTGTG                19:43373537

CD177    ENSG00000204936:     7586 GCCTCCTATACCCACTTCTGCTCCTCGGACCTGTGCAATAGTGCCAGCAGCAGCAGCGTT ENSG00000204936:     7645
                                   ||||||||||||||||||||||||||||||||||||||||||||||||||||||||||||
CD177P1                19:43373536 GCCTCCTATACCCACTTCTGCTCCTCGGACCTGTGCAATAGTGCCAGCAGCAGCAGCGTT                19:43373477

CD177    ENSG00000204936:     7646 CTGCTGAACTCCCTCCCTCCTCAAGGTATGGGATCCAGGGCCGTGGAGAAATGAGGCCCA ENSG00000204936:     7705
                                   ||||||||||||||||||||||||||||||||||||||||||||||||||||||||||||
CD177P1                19:43373476 CTGCTGAACTCCCTCCCTCCTCAAGGTATGGGATCCAGGGCCGTGGAGAAATGAGGCCCA                19:43373417

CD177    ENSG00000204936:     7706 GACACACAGAGACTCTGGCCCAAGGTGGCCAGCTGCTCTGAGCACAAAGTCATGTACCCC ENSG00000204936:     7765
                                   |||||||||||||||||||||||||||| |||||||||||||||||||||||||||||||
```

```
CD177P1               19:43373416 GACACACAGAGACTCTGGCCCAAGGTGGGCAGCTGCTCTGAGCACAAAGTCATGTACCCC              19:43373357

CD177    ENSG00000204936:    7766 CACCTTCCCTCTGCTCCCCAGCTGCCCCTGTCCCAGGAGACCGGCAGTGTCCTACCTGTG ENSG00000204936:    7825
                                  ||||||||||||||||||||||||||||||||||||||||||||||||||||||||||||
CD177P1               19:43373356 CACCTTCCCTCTGCTCCCCAGCTGCCCCTGTCCCAGGAGACCGGCAGTGTCCTACCTGTG              19:43373297

CD177    ENSG00000204936:    7826 TGCAGCCCCTTGGAACCTGTTCAAGTGGCTCCCCCCGAATGACCTGCCCCAGGGGCGCCA ENSG00000204936:    7885
                                  ||||||||||||||||||||||||||||||||||||||||||||||||||||||||||||
CD177P1               19:43373296 TGCAGCCCCTTGGAACCTGTTCAAGTGGCTCCCCCCGAATGACCTGCCCCAGGGGCGCCA              19:43373237

CD177    ENSG00000204936:    7886 CTCATTGTTATGATGGGTACATTCATCTCTCAGGAGGTGAGTGCTGCAAGCAGGGCCCCA ENSG00000204936:    7945
                                  ||||||||||||||||||||||||||||||||||||||||||||||||||||||||||||
CD177P1               19:43373236 CTCATTGTTATGATGGGTACATTCATCTCTCAGGAGGTGAGTGCTGCAAGCAGGGCCCCA              19:43373177

CD177    ENSG00000204936:    7946 AGGATGAAGGCACTGGTGGCCTGGACTCCTGGGTCTGAGGGAGGAGGGGCTGGGGGCCTG ENSG00000204936:    8005
                                  ||||||||||| |||||| ||||| |||||||| ||||||||| ||||||||||||||||
CD177P1               19:43373176 AGGATGAAGGCGCTGGTGTCCTGGGCTCCTGGGACTGAGGGAGAAGGGGCTGGGGGCCTG              19:43373117

CD177    ENSG00000204936:    8006 GACTCCTGGTCCGAGGGAGGAGGCGCTGGGGGCCT                          ENSG00000204936:    8040
                                  |||||||||
CD177P1               19:43373116 GACTCCTGG--------------------------                                        19:43373119


CD177    ENSG00000204936:    8041 GGACTCCTGGTCCGAGGGAGGAGGGGCTGGGGGCCTGGACTCCTGGTCTGAGGGAGGAGG ENSG00000204936:    8100
                                           ||| |||| |||||||||
CD177P1               19:43373118 ---------GTCTGAGGTAGGAGGGGC---------------------------------              19:43373092


CD177    ENSG00000204936:    8101 CGCTGGGGGCCTGGGCTCCTGGTCCCAGGGAGGAGGGGCTGGGTGCCTGGACTCCTGGTC ENSG00000204936:    8160
CD177    ENSG00000204936:    8161 TGAGGGAGGAGGGGCTGGGGGCCGGGGCTCCTGGGTCTGAGGAGCTGAGGCTCTGGACTC ENSG00000204936:    8220
CD177    ENSG00000204936:    8221 CTGGGTCTGAGGTAGGAGGGACTGGGGGCCTGGACTCCTGGGTCTGAGGGAGGAGGGGCT ENSG00000204936:    8280
CD177    ENSG00000204936:    8281 GGGGGCCTGGACCCCTGGGTCTGAGGAGCTGGA                            ENSG00000204936:    8313


CD177    ENSG00000204936:    8314 GCTGGGGGTCTGGGCTCCTCGGTCTGAGGGAGGAGGGTCTGGGGCCTGGACTCCTGGGTT ENSG00000204936:    8373
                                  |||||||||||| ||||||  || ||||||||||||||||||||||||||||||||||||
CD177P1               19:43373091 GCTGGGGGTCTGAGCTCCTGCGTTTGAGGGAGGAGGGTCTGGGGCCTGGACTCCTGGGTT              19:43373032

CD177    ENSG00000204936:    8374 TA-CAAC                                                      ENSG00000204936:    8379
                                  ||  ||
CD177P1               19:43373031 TATGAAT                                                                    19:43373025

CD177    ENSG00000204936:    8380 TTGGCTGGGCTGTACTCTGTGTCCTTTCTGACTTGGTCTTCTCCCTCTAGGTGGGCTGTC ENSG00000204936:    8439
                                  ||||||||||||||||||||||||||||||||||||||||||||||||||||||||| |
CD177P1               19:43373024 TTGGCTGGGCTGTACTCTGTGTCCTTTCTGACTTGGTCTTCTCCCTCTAGGTGGGCTGAC              19:43372965

CD177    ENSG00000204936:    8440 CACCAAAATGAGCATTCAGGGCTGCGTGGCCCAACCTTCCAGCTTCTTGTTGAACCACAC ENSG00000204936:    8499
                                  ||||| ||||||||||||||||| ||||||||||||||||||| ||||||||||||||||
CD177P1               19:43372964 CACCAGAATGAGCATTCAGGGCTGTGTGGCCCAACCTTCCAGCTCCTTGTTGAACCACAC              19:43372905

CD177    ENSG00000204936:    8500 CAGACAAATCGGGATCTTCTCTGCGCGTGAGAAGCGTGATGTGCAGCCTCCTGCCTCTCA ENSG00000204936:    8559
                                  ||||||||| ||||||||||||| | |||||||| |||||| || |||||||||||||||
CD177P1               19:43372904 CAGACAAATTGGGATCTTCTCTGTGTGTGAGAAGGGTGATGAGCCGCCTCCTGCCTCTCA              19:43372845

CD177    ENSG00000204936:    8560 GCATGAGGGAGGTGGGGCTGAGGGCCTGGAGTCTCTCACTTGGGGGGTGGGGCTGGCACT ENSG00000204936:    8619
                                  |||||||||||||||||||| ||||||||||| |||||||||||||||||||||||||||
CD177P1               19:43372844 GCATGAGGGAGGTGGGGCTGGGGGCCTGGAGTGTCTCACTTGGGGGGTGGGGCTGGCACT              19:43372785

CD177    ENSG00000204936:    8620 GGCCCCAGCGCTGTGGTGGGGAGTGGTTTGCCCTTCCTGCTAACTCTATTACCCCCACGA ENSG00000204936:    8679
                                  ||||||||||||||||||||||| |||||||||||||||||||||| ||| |||||||||
CD177P1               19:43372784 GGCCCCAGCGCTGTGGTGGGGAGCGGTTTGCCCTTCCTGCTAACTCCATTTCCCCCACGA              19:43372725

CD177    ENSG00000204936:    8680 TTCTTCACCGCTGCTGACCA                                         ENSG00000204936:    8699
                                  ||||||||||||||| ||||
CD177P1               19:43372724 TTCTTCACCGCTGCTCACCA                                                       19:43372705

CD177    ENSG00000204936:    8700 CCCACACTCAACC-TCCCTCTGACCTCATAACCTAATGGCCTTGGACACCAGATTCTTT- ENSG00000204936:    8757
                                  || |||||||||| |||||    | | ||||||||||||||||||||||||||||||||
CD177P1               19:43372704 CC-ACACTCAACCGTCCCTTGTGCTTG-TAACCTAATGGCCTTGGACACCAGATTATTTC              19:43372647

CD177    ENSG00000204936:    8758 CCCATTCTGTCCATGAATCATCTTCCCCACACACAATCATTCATATCTACTCACCTAACA ENSG00000204936:    8817
                                  ||||| ||||||||||||||||||||||||||| |||||||||||||||||||||||| |
CD177P1               19:43372646 CCCATCCTGTCCATGAATCATCTTCCCCACACATAATCATTCATATCTACTCACCTAATA              19:43372587

CD177    ENSG00000204936:    8818 GCAACACTGGGGAGAGCCTGGAGCATCCGGACTTGCCCTATGGGAGAGGGGACGCTGGAG ENSG00000204936:    8877
                                  ||||||||||||||||||||||||| ||||||||||||| ||||||||||||| ||||||
CD177P1               19:43372586 GCAACACTGGGGAGAGCCTGGAGCAGCCGGACTTGCCCTGTGGGAGAGGGGACACTGGAG              19:43372527

CD177    ENSG00000204936:    8878 GAGTGGCTGCATGTATCTGATAATACAGACCCTGTCCTTTCTCCCAGTGCTGGGATTTCT ENSG00000204936:    8937
                                  |||||||||||||| |||||||||| ||||||||| ||||||||||||||||||||||||
CD177P1               19:43372526 GAGTGGCTGCATGTGTCTGATAATAAAGACCCTGTGCTTTCTCCCAGTGCTGGGATTTCT              19:43372467

CD177    ENSG00000204936:    8938 CCATGTGAGGGGGCAGCAGGACACCCAGGGATCTAGCGTGGGGGAGGAGAGGAGCCTAAT ENSG00000204936:    8997
                                  |||||| ||||| ||||||||| |||||||||||||| ||| ||||||||||| ||||||
CD177P1               19:43372466 CCATGTAAGGGGACAGCAGGACGCCCAGGGATCTAGCATGGTGGAGGAGAGGAACCTAAT              19:43372407

CD177    ENSG00000204936:    8998 GAGAAAATGACCATCTAAAGCCTGCCCTTCATTGGTCTGGTTCACGTCTCCAAACCAGCT ENSG00000204936:    9057
                                  ||||||||| ||||| |||||||||||||||||||||||||||| |||||||||||||||
CD177P1               19:43372406 GAGAAAATGGCCATCCAAAGCCTGCCCTTCATTGGTCTGGTTCATGTCTCCAAACCAGCT              19:43372347

CD177    ENSG00000204936:    9058 TGGATGGTAGCAGAGACTTCAGGGTGCTCCAGCCAAACGTATTTGGGCATCACCATGACC ENSG00000204936:    9117
                                  ||||||||||||||||||||||||||||||||||||||||||||||||||||||||||||
CD177P1               19:43372346 TGGATGGTAGCAGAGACTTCAGGGTGCTCCAGCCAAACGTATTTGGGCATCACCATGACC              19:43372287

CD177    ENSG00000204936:    9118 TGGGAGGGGAAGATGCACTGAGACGTATGAGGCTTCCAGCCTAGCAGCCAGGGCCCTAGC ENSG00000204936:    9177
                                  ||||||||||||||||||||||| ||||||||||||||||||||||||||||||||||||
CD177P1               19:43372286 TGGGAGGGGAAGATGCACTGAGATGTATGAGGCTTCCAGCCTAGCAGCCAGGGCCCTAGC              19:43372227

CD177    ENSG00000204936:    9178 ACAAACAGGAGGCTCGCCCCATCTGAGCAACTGCAGGAGAGGTTAGTACAGTCATGCATT ENSG00000204936:    9237
                                  ||||||||| |||| ||||||||||||| |||||||||||||||||| ||||||||||||
CD177P1               19:43372226 ACAAACAGGGGGCTGGCCCCATCTGAGCGACTGCAGGAGAGGTTAGTATAGTCATGCATT              19:43372167
```

```
CD177    ENSG00000204936:    9238 GCTTAACGACAGGGACGTGTCGTTAGAAATGTGTCGTTAGGTGATTTTATGACCATAGGA ENSG00000204936:    9297
                                  |||| ||||||||||||||||||||||||||||||||||||||||||||| || | ||||
CD177P1                19:43372166 GCTTTACGACAGGGACGTGTCGTTAGAAATGTGTCGTTAGGTGATTTTATCACGAAAGGA             19:43372107

CD177    ENSG00000204936:    9298 ACATTGTAGCGTGCACTTACACCAACCCAGATGGTACAGCCCAATACACACCCAGGATGG ENSG00000204936:    9357
                                  ||||||||| ||||||||||||||||||||||||||||||||||||||||||||||||||
CD177P1                19:43372106 ACATTGTAGAGTGCACTTACACCAACCCAGATGGTACAGCCCAATACACACCCAGGATGG             19:43372047

CD177    ENSG00000204936:    9358 ACGCTAGAGTCGACTGCTCCTAGGCTACAAGCCTGCAGTGCATGTTATGGTGTGAATACT ENSG00000204936:    9417
                                  | | |||||| |||||||||||||||||||||||||||||||||||||||||||||||||
CD177P1                19:43372046 ATGGTAGAGTTGACTGCTCCTAGGCTACAAGCCTGCAGTGCATGTTATGGTGTGAATACT             19:43371987

CD177    ENSG00000204936:    9418 GCAGGCAA                                                     ENSG00000204936:    9424
                                  |||||||
CD177P1                19:43371986 GCAGGCA-                                                                 19:43371980

CD177    ENSG00000204936:    9426 TCTTAACACCACGGCAAGTATTTGTGCATCTACACATCTAAACATAGAAAAGGTACAG ENSG00000204936:    9485
                                  || ||||||||||||||||||||||||||||| |||||||||||||||||||||||||||
CD177P1                19:43371979 TCGTAACACCACGGCAAGTATTTGTGCATCTATACACATCTAAACATAGAAAAGGTACAG             19:43371920

CD177    ENSG00000204936:    9486 CATAAATACACTATTGTCATCTCAGCAGA                                ENSG00000204936:    9514
                                  ||||||||||||||||||||||||| |||
CD177P1                19:43371919 CATAAATACACTATTGTCATCTCAGGAGA                                            19:43371891
```
